# Validation and optimization of a multi-processor UV-C reactor for inactivation of potato brown rot and soft rot pathogens in surface irrigation water in the Nile Delta

**DOI:** 10.1038/s41598-026-67661-0

**Published:** 2026-09-09

**Authors:** Fathi A. A. Hassan, Ashraf F. Abd El-Rahman, Kamel M. A. Elhalag, Ahmed E. Azab, Nevein A. S. Messiha

**Affiliations:** 1 Biological Engineering Department, Agricultural Engineering Research Institute, Agricultural Research Center (ARC), Dokki, Giza, Egypt; 2 Bacterial Diseases Research Department, Plant Pathology Research Institute, Agricultural Research Center (ARC), 12619 Giza, Egypt

**Keywords:** Irrigation water quality, UV-C disinfection, *R*. *solanacearum*, *P*. *carotovorum*, Bacterial plant diseases control, Biotechnology, Environmental sciences, Microbiology, Plant sciences

## Abstract

Bacterial wilt and soft rot, caused by *Ralstonia solanacearum* and *Pectobacterium carotovorum*, are major threats to potato production and export. Surface irrigation water (SIW) can transmit these pathogens. This study validated and optimized a multi-processor UV-C (MP UV-C) reactor to ensure effective pathogen inactivation while minimizing energy use and operational cost. Physicochemical analysis revealed moderate turbidity and total suspended solids (TSS) in SIW, with UV transmittance (UVT) levels below the optimal range. This necessitates pre-filtration. Passing water through a 5-micron sediment filter improved UVT from 75% to 85%, reduced turbidity from 7.82 to 2.7 nephelometric turbidity units, and lowered TSS, chemical oxygen demand, and total dissolved solids, thereby placing the water within recommended ranges for UV-C disinfection. Collimated beam tests established UV dose–response curves, showing strong polynomial correlations (R^2^ > 0.99) between the applied UV dose and log inactivation. *R*. *solanacearum* exhibited higher UV sensitivity (6.23 mJ/cm^2^/log) than *P*. *carotovorum* (10.30 mJ/cm^2^/log). Reactor optimization identified an operational optimal setting of 270 W lamp power at a 35 m^3^/h flow rate, delivering 52.54 mJ/cm^2^ and achieving a 4-log reduction in both pathogens. Scanning electron microscope (SEM) analysis of *R*. *solanacearum* revealed severe cell wall damage at higher UV doses. Bioassays confirmed that UV-C-treated SIW significantly suppressed bacterial wilt in tomato seedlings and soft rot in potato tubers. Future work should assess the large-scale economic feasibility of UV-C treatment and its long-term effects on soil microbial biodiversity to ensure both sustainability and microbial balance in agricultural systems.

**Supplementary Information:**

The online version contains supplementary material available at https://doi.org/10.1038/s41598-026-67661-0.

## Introduction

After rice, wheat, and maize, potato (*Solanum tuberosum*) is the world’s fourth most important crop^1,2^. Its high-calorie, vitamin, and mineral content promotes food security for millions of people. It is a valuable cash crop that contributes significantly to both domestic and international trade, with annual output exceeding 374 million tons across 150 nations. It tolerates a wide range of climatic and soil conditions. Potato is Egypt’s most important cash crop, with production exceeding 6.87 million tons (FAO^3^). The country ranks 12th in global ware potato production and is the world’s sixth-largest exporter. The value of potato exports reached $195 million in 2021^4^. In 2023, Egypt shipped a record 440,000 tons of potatoes to EU countries. This illustrates the significance of potatoes to the economy and the global demand for them^5^. Moreover, Egypt’s potato exports for the 2023-24 season totaled approximately 650,000 tons to the European Union, Russia, and Arab countries, according to Egyptian news reports.

Bacterial diseases are among the world’s most significant biotic constraints on potato production. Bacterial wilt, caused by *R*. *solanacearum*; soft rot and blackleg, caused by *Pectobacterium* spp. and *Dickeya* spp.; ring rot, caused by *Clavibacter sepedonicus*; common scab, caused by *Streptomyces* spp, and zebra chip, caused by *Candidatus Liberibacter solanacearum*, are the most critical bacterial diseases affecting potatoes. *R. solanacearum* is one of the top ten most destructive plant pathogenic bacteria worldwide due to its broad host range, long environmental survival period, and aggressiveness^6^. Farmers worldwide are suffering from brown rot, which causes annual losses exceeding $950 million^7^. Moreover, international phytosanitary regulations prohibit the export of contaminated consignments, further pressuring farmers’ incomes and affecting national economies. In Egypt, the disease results in annual losses due to rejected consignments and decreased productivity. *R*. *solanacearum* survives in soil, water, and plant waste and can disseminate through irrigation systems.

Different traditional disease management strategies are being implemented, including crop rotation^8,9^, resistant varieties^10^, biological soil disinfestation^11^, natural compounds^12^, and biocontrol agents^13^. The traditional disease management strategies have shown limited and inconsistent success. Furthermore, biocontrol agents frequently encounter obstacles with establishment, consistency, and efficiency in field and large-scale conditions. The use of some chemical pesticides often has limited effectiveness, and their application can lead to significant environmental damage, promote the emergence of resistant bacterial strains, and disrupt the balance of local microbial communities^14^. Furthermore, these approaches primarily ignore the essential aspect of waterborne disease transmission.

Water is considered the primary input in agricultural systems; therefore, the quality of irrigation water significantly impacts agricultural output. Irrigation water is regarded as a key factor in the spread and invasion of the pathogen to new areas^15^. Contaminated SIW, canals, drains, and rivers containing *R. solanacearum* and *P*. *carotovorum* provide an ideal medium for the spread of these pathogens, facilitating long-distance infection^16,17^. Studies have confirmed that SIW in the Nile Delta is frequently contaminated with these pathogens, which can persist for weeks under favorable environmental conditions^18^. This spread through irrigation water is a primary challenge to controlling bacterial wilt and other waterborne pathogen outbreaks, indicating the urgent need for efficient water treatment. Because irrigation water is a continuous and essential source of many pathogens, disinfection is a vital step toward disease management^19^. Conventional water disinfection treatments, such as chlorination and ozonation, can produce toxic byproducts, be expensive, or be unfeasible for agricultural use^20–22^. These highlight the urgent need for novel and convenient water treatment technologies that farms can readily adopt.

UV-C irradiation is being recognized as an effective and environmentally safe physical disinfection technology that can inactivate a wide range of microorganisms, including bacterial waterborne pathogens. UV-C light, with wavelengths ranging from 200 to 280 nm, exhibited antimicrobial activity primarily by destroying microbial DNA and RNA through the formation of pyrimidine dimers, mainly thymine-thymine and cytosine-thymine dimers, which inhibit replication and transcription and consequently lead to microbial inactivation^23,24^. UV-C doesn’t leave undesirable residues or alter water chemistry, making it an ecologically safe option for irrigation water treatment^25^. Studies indicate that gram-negative bacterial genera such as *Ralstonia* and *Pectobacterium* are more susceptible to UV-C inactivation than gram-positive bacteria and fungi, which tend to be more resistant to UV-C and often require higher doses or additional treatment steps^26^. The UV-C dose, expressed as the product of light intensity (mW/cm²) and exposure time (seconds) and commonly reported in mJ/cm², is a critical parameter governing the disinfection efficiency^27^. The required dose depends on the microorganism’s resistance, water quality parameters, and the configuration of the UV-C reactor and operational conditions^28–30^. Water quality factors, such as ultraviolet transmittance (UVT), turbidity, and suspended solids, can significantly impact UV penetration and cause shielding of pathogens, thereby reducing inactivation efficiency^31^. Similarly, hydraulic flow rate and reactor geometry influence exposure uniformity and the actual dose delivered to microorganisms.

Despite growing interest in UV-C applications, field studies focusing specifically on phytopathogenic bacteria in irrigation systems remain limited, especially in developing countries. The majority of existing research addresses human pathogens in drinking water, with fewer investigations targeting agricultural pathogens in SIW^32^. This knowledge gap limits the practical application of UV-C technology in irrigation water treatment for controlling plant disease. Accordingly, the central hypothesis of this study is that UV-C irradiation can be effectively implemented in a field-compatible manner to disinfect SIW, thereby reducing populations of *R*. *solanacearum* and *P*. *carotovorum* and consequently decreasing the incidence of bacterial wilt and soft rot in potatoes grown in the Nile Delta region of Egypt. To achieve this, the research aims to (i) determine the sensitivity of both pathogens, *R*. *solanacearum* and *P*. *carotovorum*, to UV-C light by deriving a dose-response curve (DRC) under laboratory conditions; (ii) validate the ability of the proposed MP UV-C reactor for irrigation water disinfecting to deliver the required doses to inactivate target pathogens and its effect on their pathogenicity; and (iii) determine the optimum operating conditions (flow rate, lamp power combination) for achieving the maximum disinfection rate.

## Materials and methods

### Description of the MP UV-C reactor

**Fig. 1:**
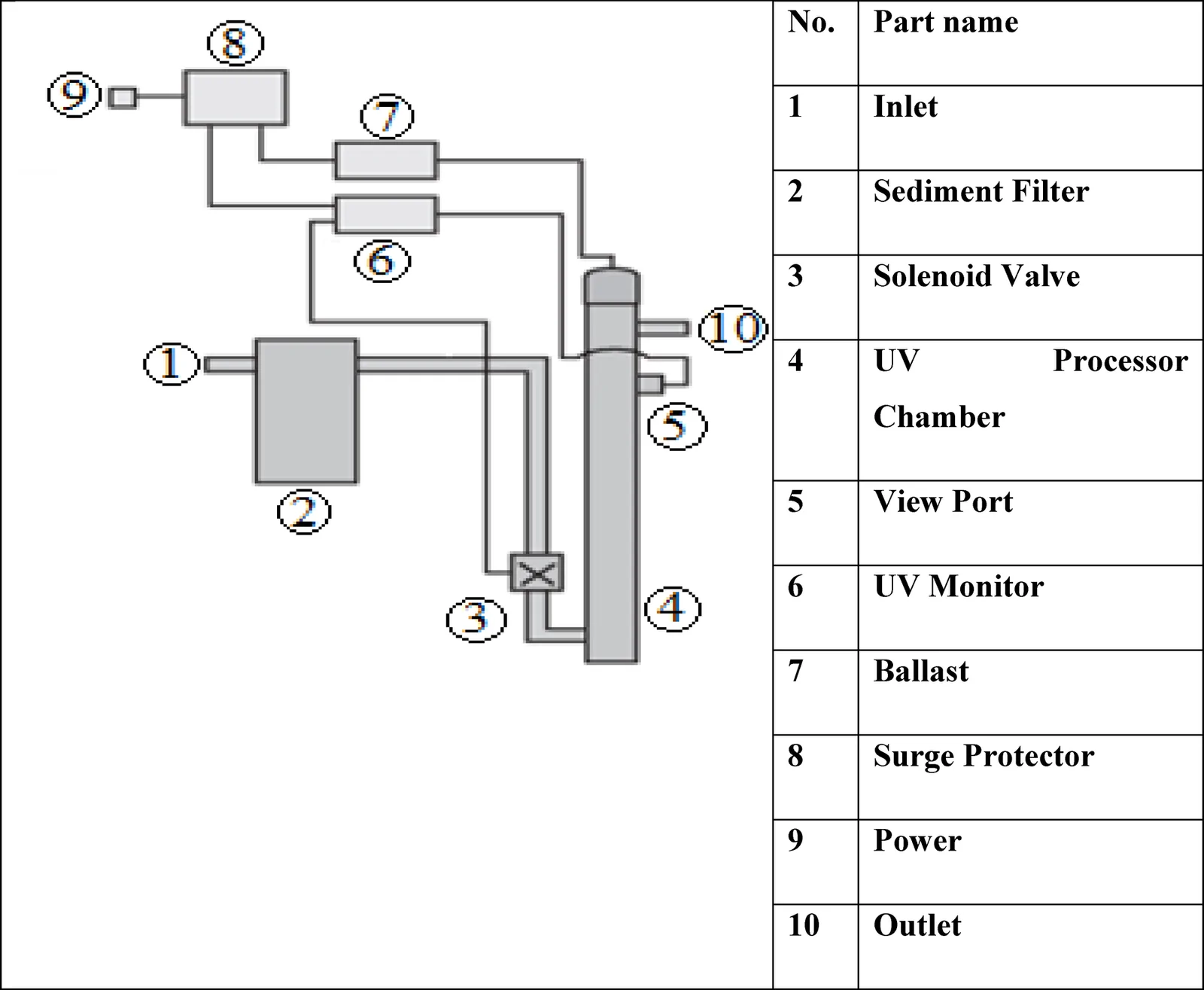
Schematic diagram of the vertical installation of the UV-C processor.

The proposed MP UV-C reactor was designed and constructed by Alex Power for General Supplies & Trade (Matrouh, Egypt). The design was developed according to specifications established through reviewing prior publications^33^ and validated by preliminary tests. The most important design criteria considered were a flow rate of at least 35 m³/h, water transmittance of ≥ 65%, an inactivation level of > 4 log, and a UV dose of ≥ 100 mJ/cm². The proposed reactor had to be multiprocessor, with a total combined power of the UV lamps exceeding 300 W. The UV lamp output power had to be adjustable to facilitate the inactivation of a wide range of plant pathogens. The proposed UV-C reactor, called the MP UV-C reactor, consists of nine UV-C processors installed vertically in parallel. The peak wavelength of the UV lamp in all processors was 253.7 nm. Supp. Figure 1 illustrates the MP UV-C reactor and its operation in the laboratory and in the field. Figure 1 shows a schematic diagram of the vertical installation of the UV-C processor. Supp. Figure 2 illustrates a detailed diagram of the UV-C processor along with a list of its parts. Supp. Table 1 lists the technical specifications of the MP UV-C reactor. Supp. Table 2 also shows the geometric dimensions of the housing, quartz sleeve, and UV-C lamp data sheet for each processor.

**Table 1:** Validation parameters for *Ralstonia solanacearum* and *Pectobacterium carotovorum* isolates.

Isolate	Parameter	log reduction (CFU//mL water)								Average
		0.5	1	1.5	2	2.5	3	3.5	4	
*R*. *solanacearum*	RED	2.76	5.72	8.89	12.26	15.83	19.62	23.6	27.79	-
	Sensitivity	5.52	5.72	5.93	6.13	6.34	6.54	6.74	6.95	6.23
	D_Val_	2.42	5.02	7.79	10.75	13.89	17.21	20.71	24.38	-
*P*. *carotovorum*	RED	3.95	8.60	13.9	19.90	26.62	34	42.1	50.83	-
	Sensitivity	7.9	8.60	9.27	9.95	10.65	11.33	12.03	12.71	10.30
	D_Val_	3.46	7.54	12.19	17.45	23.35	29.82	36.93	44.59	-

RED = Reduction equivalent dose (mJ/cm²), D_Val_ = Validated dose (mJ/cm²).

**Table 2:** Calculated available UV-C Dose (mJ/cm²) under different flow rates and lamp power combinations during multi-processor UV-C reactor optimization.

Lamp power (W)	Flow rate (m^3^/h)					
	15	20	25	30	35	40
150	64.27 *	48.20*	38.56**	32.14**	27.54**	24.10***
210	93.43*	70.08*	56.06*	46.72*	40.04**	35.04**
270	122.60*	91.95*	73.56*	61.30*	52.54*	45.97*
330	151.76*	113.82*	91.06*	75.88*	65.04*	56.91*

* Dose disinfected both *Ralstonia solanacearum* and *Pectobacterium carotovorum*;.

** Only *R*. *solanacearum* disinfected; *** None.

A set of filters was used to reduce the irrigation water turbidity to a level that allows the use of UV for water disinfection: The filter set consisted of 1. Screening filter.

2. Disk filter. These are fixed/installed before the pump to purify the water down to 0.5 mm. 3. Microfiber filter: It is installed before the UV unit to reduce water turbidity to 5 microns. To maintain proper operating pressure, a suitable pump was selected to meet the required flow rate.

Filters are maintained when a change in flow rate is observed. Maintenance may involve cleaning or replacing the first and second filters, or replacing the third filter.

### Surface irrigation water source and sampling

SIW was obtained from a branch canal of a main canal connected to the Nile River, located in Kafr Shukr (30°34’02.0” N 31°14’51.9” E) in the Qalyubia Governorate, Egypt. This area is known for occurrences of bacterial potato diseases, including bacterial wilt^9^. Water samples were collected using an irrigation pump from a depth of approximately 10–20 cm below the water surface. SIW was collected and stored in 500 L plastic tanks for seven days to accumulate sufficient volume for testing. The natural load of target pathogens in irrigation water was quantified prior to experimental work, following the method of Wenneker et al.^34^.

### Water analysis

Two composite water samples were prepared. One was collected before filtration, and the other after filtration through a 5-micron filter mounted on the proposed MP UV-C reactor. Each composite sample was obtained by homogenizing three SIW subsamples. The samples were then sent to the Agricultural Engineering Research Institute (AEnRI), ARC, Giza, Egypt, for analysis. There, pH was measured using the pH100 ECO sense meter; electrical conductivity (EC) using the EC 300 ECO sense meter; total dissolved solids (TDS) using TDS-3 and TDS/Temp; TSS according to the standard method (APHA et al.^35^); total alkalinity; and total hardness (CaCO₃ mg/L) according to standard methods (APHA et al.^36^). Turbidity was also measured using the PCE-TUM 20 portable turbid meter. Turbidity values were recorded in nephelometric turbidity units (NTU). A UVA, UVC light meter Model YK-37UVSD was utilized to determine the UVT% (Berson, the Netherlands). The procedure provided by the manufacturer was followed, and the photometer was calibrated using distilled water, which represented a UVT% of 100%. The key elements iron (Fe) and manganese (Mn), along with chemical oxygen demand (COD) and total organic carbon (TOC), were measured in the water analysis laboratory at the Regional Center for Food and Fodder (RCFF), ARC, Giza, Egypt. Sample collection, inspection, preservation, and analyses were performed according to standard methods (APHA et al.^37^).

### Bacterial strains and growth conditions

*R*. *solanacearum* strain STDF2905 (Acc. No. OR430097) and *P*. *carotovorum* strain 100 H (Acc. No. OP603322.1) were used in this study. These two pathogens were originally isolated by the co-authors, NAS Messiha and KMA Elhalag, from symptomatic potato plants exhibiting bacterial wilt and soft rot, respectively^38,39^. The virulence of *R. solanacearum* was confirmed by stem inoculation of tomato seedlings^40^, which resulted in pronounced wilting symptoms. Conversely, the virulence of P. carotovorum (100 H) was established through its ability to induce tissue maceration and soft rot in potato tubers. R. solanacearum and P. carotovorum were cultured separately on 150 mm diameter nutrient agar plates and incubated at 28 °C for 48 h. Bacterial cells from each strain were harvested and re-suspended in separate experimental water samples. The concentration of each bacterium in the experimental water was adjusted to an optical density at 600 nm (OD600) of 0.1 using a UNICO 2000 spectrophotometer, corresponding to approximately 10^8^ CFU/mL^41^.

### Detection of the target pathogens

Detection of R. solanacearum was made using the semi-selective SMSA medium^42^, followed by indirect immunofluorescent-antibody staining (IFAS)^40^ and/or conventional PCR using specific primers (Rs-1-F/5`-ACTAACGAAGCAGAGATGCATTA-3`; R/5`CCCAGTCACGGCAGAGACT-3) according to^43^. Detection of P. carotovorum was made using Logan medium^44^, followed by conventional PCR. Oligonucleotide primers EXPCCR (5′-GCCGTAATTGCCTACCTGCTTAAG-3′) and EXPCCF (5′-GAACTTCGCACCGCCGACCTTCTA-3′) were employed in a standard PCR assay^45^.

### Realistic validation

Validation testing is conducted according to USEPA^46^ to verify the reactor’s performance and establish the range of acceptable operating conditions. This ensures the proposed MP UV-C reactor can effectively deliver the dose required for ≥ 4-log inactivation of *R*. *solanacearum* and *P*. *carotovorum* pathogens in irrigation water. Using actual target pathogens provides realistic performance data for the UV-C reactor under conditions relevant to agricultural water treatment in the Nile Delta, Egypt. The validation procedure, known as biodosimetry, determines the log inactivation for a specific pathogen and relates it to the operating conditions at the time of testing (e.g., UVT, flow rate, and lamp power combinations). Reactor validation included two complementary experimental approaches: collimated beam tests and biodosimetry tests.

### Collimated beam test

The collimated beam (CB) test is the initial step in validating the performance of the UV-C reactor. It measures the appropriate laboratory dose to achieve a desired reduction level for a particular microbe under specific operating conditions. The CB tests were performed in accordance with the ‘Ultraviolet Disinfection Guidance Manual for the Final Long Term 2 Enhanced Surface Water Treatment Rule’ (UVDGM LT2ESWTR) (USEPA^47^, complemented with the UVDGM Proposal Draft (USEPA^46^. The CB tests were conducted in the laboratory of the Bacterial Diseases Research Department at the Plant Pathology Research Institute. The CB apparatus was manufactured using a simplified design modified by Bolton et al.^48^, as shown in Supp. Figure 3a. This apparatus depends on a low-pressure (LP) mercury vapor UV lamp (TUV T5 Phillips) with a nominal power of 6 W and an arc length of 22 cm as a source of a collimated UV beam. The distance of the UV lamp from the Petri plate was fixed at 25 cm to ensure uniform radiation through the sample surface. The CB apparatus produces a precise, uniform UV-C light at a wavelength of 253.7 nm, which is used to determine the UV DRC of the target pathogens *R*. *solanacearum* and *P*. *carotovorum*. Before each treatment, the UV intensity “mW/cm^2^” delivered by the CB with no sample present was measured using a calibrated UVA/UVC light meter, Model YK-37UVSD, as shown in Supp. Figure 3b. The average value of light intensity for all treatments was taken as 0.124 mW/cm². A CB test on a bench scale was performed for the highest and lowest UVT conditions during the entire test. At the beginning of each biodosimetry test day, one liter was collected from the water tank prior to pumping into the UV-C reactor to validate each test condition, and prepare samples for CB assays.

For each CB test,10 mL of a water sample was placed into a sterilized Petri dish (52.4 mm inner diameter x 14 mm deep) containing a stir bar. Water samples were continuously agitated at 60 rpm using a magnetic stirrer. The Petri dish was positioned 10 mm away from the end of the collimating tube. Samples were exposed to specific doses of CBs of 5, 10, 20, 30, 40, 50, 60, 70, 80, 90, and 100 mJ/cm² for *P*. *carotovorum* samples and 2.5, 5, 10, 15, 20, 25, 30, 35, 40, 45, and 50 mJ/cm² for R. solanacearum. Supp. Table 3 lists the UV dose applied by CB and the corresponding exposure time for each pathogen.

Once the mixing was initiated, the cardboard shutter was removed, and the irradiation timer was started simultaneously. After the specific exposure time, the cardboard was reinserted between the collimating tube and the Petri dish. Each treatment was in triplicate. Following irradiation, the Petri dish was removed, and the sample was collected for the quantification of the challenge microorganism concentration. The samples were stored in the dark at 4 °C for bioassay.

UV doses delivered to the water samples were calculated according to UVDGM (USEPA^47^, following the standardized protocol described by Bolton et al.^49^,, considering the different correction factors (reflection, Petri, water, and divergence factor), using Eq. 1:

1 $${D_{CB~}}=~{E_s} \times {P_f} \times \left( {1 - R} \right) \times \frac{L}{{d+L}} \times \frac{{~1 - {{10}^{\left( { - {A_{254~~}} \times d} \right)}}}}{{{A_{254~}}~ \times ~d~ \times ~\ln \left( {10} \right)}} \times t$$

Where D_CB_ = UV dose (mJ/cm^2^); E_s_ = average UV intensity (measured before and after irradiating the sample) (0.124 mW/cm^2^); P_f_ = Petri factor (0.9); R = reflectance at the air-water interface at 254 nm (≈ 0.025); L = distance from the lamp centerline to the water surface (25 cm); d = depth of the suspension (0.46 cm); A_254_ = absorbance at 254 nm, calculated as -log_10_(UVT); let UVT = 0.75, then A_254_ = 0.125; and t = exposure time (s), calculated based on the desired UV dose using Eq. (1). The culturable colony-forming units (CFU) per mL for each replicate were calculated as the average across the three replicates.

### Development of the UV dose-response curve

Collimated beam (CB) tests produce UV doses expressed in mJ/cm², while the concentration of microorganisms in the Petri dish before and after UV exposure was represented by N₀ and N, respectively, in CFU/mL. Full-scale reactor testing was conducted over three days, with one UV dose-response curve (DRC) developed for each UVT condition per day. UV DRCs were developed following USEPA^47^ guidelines.

For each test condition and replicate, log N was plotted against UV dose to identify a common N0, taken as the intercept of the curve at UV dose = 0. Log reduction (log I) is then calculated for each measured value of N, including zero-dose, using the identified common N0 according to Eq. 2:

2 $$Log~reduction~\left( {\log ~I} \right)={\log _{10}}\frac{{{N_0}}}{N}=\log {N_0} - \log N$$

Where N_0_ and N represent the concentration of infectious pathogen before and after exposure to UV light, respectively. The average log inactivation for three replicates was calculated to produce one value of log I per test condition. The UV dose is then plotted as a function of log I for each test condition, and regression analysis is used to derive the equation that best fits the data, ensuring the fit passes through the origin. The forms of the equation vary depending on the data.

The goodness-of-fit of the equation is evaluated by examining whether the differences between measured UV dose values and those predicted by the equation are randomly distributed around zero and independent of UV dose. Significance of regression coefficients was assessed using p-values, with α = 0.05 as the threshold for statistical significance. CB data are often fit to a polynomial regression. The 95-percent confidence interval (U_DR_) is estimated using Eq. 3:

3 $${U_{DR}}=t~ \times \frac{{SD}}{{U{V_{dose~CB}}}}~ \times 100\%$$

Where:

U_DR_ represents the uncertainty of the UV dose-response fit at a 95-percent confidence level, UV_dose CB_ is the UV dose calculated from the UV DRC for each pathogen, SD is the standard deviation of the difference between the calculated UV dose response and measured value, and t is the t-statistic at a 95% confidence, corresponding to the number of replicates used (36 replicates are used, t equals 2.04, per USEPA^47^.

### Biodosimetry testing

Full-scale biodosimetry tests of the (MP) UV-C reactor were conducted at the Irrigation Systems and Equipment Testing Laboratory, Agricultural Engineering Research Institute (AEnRI), Egypt, following the USEPA^46^ recommended protocol. Supplementary Fig. 4 presents a block diagram of the biodosimetry test facility. The UVDGM validation framework was applied using the calculated UV dose approach for reactor performance assessment.

On each experimental day, laboratory-cultured strains of *R. solanacearum* and *P. carotovorum* were prepared, and the bacterial suspensions were adjusted by serial dilution and spectrophotometric estimation, followed by confirmation using colony-forming unit (CFU) enumeration on selective media. The stored irrigation water in the tank was then spiked with the prepared inoculum to achieve a standardized initial concentration of approximately 10^8^ CFU/mL (8 log10 CFU/mL) for both target pathogens. This inoculum level was verified before each experimental run to ensure uniform starting conditions across all treatments, including control (uninfected water), untreated inoculated water, and UV-C treated water.

The prepared inoculated water was pumped into the MP UV-C reactor for forty-eight experimental runs plus controls, covering flow rates of 25, 30, 35, and 40 m³/h, lamp power combinations of 150, 210, 270, and 330 W, and UVT levels of 75, 85, and 95%. Log inactivation was determined by comparing pre‑ and post‑treatment bacterial counts to verify that reactor performance achieved or exceeded the target doses established through CB testing.

Supplementary Table 4 summarizes the coded experimental variables, and Supplementary Table 5 presents lamp number and wattage combinations.

### Reduction equivalent dose

Because the actual UV dose cannot be measured directly in the full-scale UV-C reactor, the reduction equivalent dose (RED) was calculated indirectly by interpolating the log inactivation results from the full-scale reactor testing onto the UV DRCs from the CB testing. RED values are specific to the challenged microorganism used during CB testing and the validation test conditions for full-scale reactor testing. The validation testing for the proposed reactor was done with the two target pathogens, *R*. *solanacearum* and *P*. *carotovorum*. The UV sensitivity of the target plant pathogens was determined for each test *condition by using* Eq. 4:

4 $$UV~sensitivity~\left( {mJ/c{m^2}/\log ~1} \right)=\frac{{RED}}{{\log ~I}}$$

Where RED = The RED for the test replicate as derived by inputting log I into the UV dose-response equation, Log I = log inactivation for the test replicate (calculated previously).

### Validated reduction equivalent dose

The RED value is adjusted for experimental uncertainties and biases using a pathogen-specific validation factor of safety (VF) to produce a pathogen-specific validated dose (D_Val_). The 2006 UVDGM states that the validated reduction equivalent dose or required dose is given by Eq. 5:

5 $${D_{Val}}~\left( {mJ/c{m^2}} \right)=\frac{{RED}}{{VF}}$$

Where VF = Validation Factor of safety given by Eq. 6:

6 $$VF~=~{B_{RED}} \times \left( {1+\frac{{~{U_{Val}}}}{{100}}} \right)$$

Where B_RED_ = RED Bias Factor calculated as the ratio of the test microbe RED to the pathogen RED (since the challenging microorganism is the same as the target pathogen, then B_RED_ = 1); U_Val_ = Uncertainty of Validation expressed as a percentage of the RED, calculated using Eq. 7:

7 $${U_{Val}}={(U_{{DR}}^{{~~~~~2}}+~U_{S}^{{~~2}}+~U_{{in}}^{{~~~2}})^{0.5}}$$

Where U_DR_ = uncertainty of the UV dose-response fit calculated by Eq. 3; U_S_ = uncertainty of the UV sensors value, expressed as a fraction; determined by summing the uncertainties that arise from calibration (U_1_), linearity (U_2_), temperature response (U_3_), angular (U_4_), spectral (U_5_), and long-term stability (U6) by using Eq. 8:

8 $${U_S}=uc~ \times ~k~$$

Where k = coverage factor at 95% confidence level = 2 and uc = total standard uncertainty calculated by Eq. 9:

9 $$uc=\sqrt {u_{1}^{2}+u_{2}^{2}+u_{3}^{2}+u_{4}^{2}+u_{5}^{2}+u_{6}^{2}}$$

Where u_1_, u_2_, u_3_, u_4_, u_5_, and u_6_ are the standard uncertainties from each identified source: 1, 3, 3, 3, 2, and 4; U_in_ = Uncertainty of interpolation, calculated using Eq. 10:

10 $${U_{in}}=\frac{{t \times SD}}{{RED}} \times 100\%$$

SD = Standard deviation of the differences between the RED (based on the observed log inactivation and UV DRC) and the RED calculated using the dose-monitoring equation for each replicate; RED = The RED as calculated using the dose-monitoring equation; t = t-statistic at a 95-percent confidence level for a sample size equal to the number of test conditions used to define the interpolation according to USEPA^47^; as the test condition = 16, then t = 2.12.

### Optimization performance of the MP UV-C reactor

Although UV disinfection offers health and environmental advantages over chemical disinfectants, it remains relatively energy-intensive. Enhancing energy efficiency is therefore essential for the sustainable application of the technology. To identify the optimum configuration of the MP UV-C reactor, biodosimetry data were analyzed to determine the flow rate and lamp power combination that delivered the validated UV dose required for a 4-log (> 99.99%) inactivation of *R*. *solanacearum* and *P*. *carotovorum* with the lowest energy consumption. Average UV doses from individual processors and cumulative doses from processor sets were calculated and compared with the validated pathogen-specific doses. This enabled the selection of operating conditions that ensured reliable dose delivery at the highest feasible flow rate with minimum energy use. Available UV-C doses under different operating conditions (flow rate, lamp power, and UVT) were calculated using the UV-C dose calculation utility developed by Song Phung^50^; based on lamp intensity, exposure time, using lamp nominal power and reactor dimensions (length and diameter) of (lamp, quartz sleeve, and chamber) (Supp. Table 2), also incorporates lamp aging and fouling factors. The calculated doses were verified through manual calculations using standard formulas.

### Bioassay for evaluating the effect of irrigation water treatment by using the MP UV-C reactor on culturable microbial diversity

The experiment was designed to evaluate the effect of filtration and UV-C irradiation on the total number of culturable bacteria in SIW. The MP UV-C reactor was operated under optimal conditions. SIW passed through the MP UV-C reactor without filtration and without UV-C irradiation served as the control treatment. For the filtration treatment, SIW was passed through the filtration unit (5-micron filter) to increase UVT from 75% to 85%. For the UV-C treatment without filtration, unfiltered SIW (75% UVT) () was exposed to UV-C irradiation. For the combined treatment, the SIW was filtered to achieve 85% UVT and subsequently exposed to UV-C irradiation. The total number of culturable bacteria was determined by plating on nutrient agar (NA), and the decrease in bacterial density was calculated as the log₁₀ difference between the pre- and post-treatment levels. DNA extraction was performed on UV-resistant bacterial colonies using the DNA Easy Pure Genomic DNA Kit Trans (code #EE10101), followed by DNA sequencing, as described by Messiha et al.^9^.

### Effect of UV-C doses on *R. solanacearum* cell wall

*R. solanacearum* was grown on NA and incubated at 28 °C for 2 days. The pathogen cells were suspended in sterile PB0.01 M and standardized spectrophotometrically to 10^8^ CFU/mL. Samples are exposed to specific doses of CBs of 25 and 50 mJ/cm² using the CB apparatus. The samples were centrifuged at 10,000 rpm. A fixative solution (2.5% glutaraldehyde and 2% paraformaldehyde in 0.1 M sodium phosphate buffer, pH 7.4) was applied to the sample at a volume at least double that of the sample. The mixture was incubated at 4 °C overnight, then washed three times for 15 min in 0.1 M sodium phosphate buffer and 0.1 M sucrose before being post-fixed in 2% sodium phosphate-buffered osmium tetroxide (pH 7.4) for 90 min. After post-fixation, samples were washed three times for 15 min each with 0.1 M sodium phosphate buffer. Dehydration was performed sequentially: two cycles in 50% ethanol (15 min each), overnight incubation in a contrast solution (70% acetone, 0.5% uranyl acetate, 1% phosphotungstic acid at 4 °C), two cycles in 80% ethanol, two in 90% ethanol, two in 96% ethanol (15 min each), and three immersions in 100% ethanol (20 min each). Samples were coated with gold-palladium membranes and examined using a Jeol JSM-6510 L.V SEM. The SEM operated at 30 kV with a magnification of 5000×. The stage was positioned at X = 12.860, Y = 3.839, Z = 14.000, T = 0.000, and *R* = 0.000, with a spot size of 33. Imaging was performed at the Electron Microscopy Unit, Mansoura University, Egypt.

### Bioassay for evaluating the effect of irrigation water treatment by using the MP UV-C reactor on bacterial wilt

A bioassay was conducted in an open greenhouse facility of the Plant Pathology Research Institute to assess the actual efficiency of the MP UV-C reactor in suppressing *R*. *solanacearum*, the causal agent of bacterial wilt (brown rot), by disinfecting SIW. Tomato (*Solanum lycopersicum*) seedlings (cv. Pinto), 2–3 weeks old, were planted in sterilized plastic pots (20 cm diameter) containing a sterile soil mixture (sand-loamy: compost, 2:1). Three sets of pots were used, with twenty pots per set and two tomato plants per pot. The first set was irrigated with sterilized SIW as a negative control. The second set was irrigated with SIW contaminated with *R*. *solanacearum* (10^8^ CFU/mL) as a positive control. The third set was irrigated with SWI after passing the irrigation water, which was artificially contaminated with *R*. *solanacearum* (10^8^ CFU/mL), through the MP UV-C reactor under optimal disinfection conditions. The experiment was repeated twice, each lasting 32 days. Disease incidence was measured by the area under the disease progress curve (AUDPC). AUDPC was calculated based on the percentage of wilted leaves per pot (plant) over time^51^

11 $$Disease\,index~\left( \% \right)=~\frac{{\sum \left( {disease~rating \times ~~EquationNumber~of~plants~representing~each~disease~rating} \right)}}{{\left( {total~~EquationNumber~of~plants~ \times highest~disease~rating} \right)}}~ \times 100~$$

Disease rating was calculated as follows: 0 = no leaves wilted,1 = one leaf wilted, 2 = two or three leaves wilted, 3 = most leaves wilted, 4 = all leaves wilted, and 5 = plant dead^52^. Positive and negative wilt incidence was confirmed by IFAS^40^.

### Bioassay for evaluating the effect of irrigation water treatment by using the MP UV-C reactor on bacterial soft rot

An *in planta* bioassay was conducted to evaluate the effectiveness of the MP UV-C reactor in inactivating *P*. *carotovorum* in artificially infected irrigation water, thereby suppressing the potato soft rot caused by the pathogen in potato tubers. Tubers of the Monalisa cultivar, kindly provided by the Virus and Phytoplasma Research Department, Plant Pathology Research Institute, ARC, Giza, Egypt, were used in this experiment. Before inoculation, the tuber surfaces were disinfected with 1% (v/v) sodium hypochlorite for 10 min, rinsed with sterile distilled water (SDW), and dried with sterile tissue paper. A cylindrical well (8 mm in diameter and 15 mm deep) was created in each tuber using a sterilized cork borer. Three sets of treatments were performed, with eight potato tubers done for each treatment. For the first group, 50 µL of irrigation water was taken and pumped into the well made in the potato tuber after passing the irrigation water, which was artificially contaminated with *P*. *carotovorum* (10^8^ CFU/mL), through the MP UV-C reactor under optimal disinfection conditions. For the second set, 50 µL of UV-untreated irrigation water contaminated with *P*. *carotovorum* (10^8^ CFU/mL) was applied to the well of the tuber as a positive control, while for the third set, 50 µL of sterilized SIW was also applied to the well of the tuber as a negative control. Three replicates were used for each treatment. The removed tuber plugs were replaced, and the wounds were sealed with molten sterile paraffin wax. The inoculated tubers were placed in plastic boxes lined with moistened filter papers and incubated at room temperature for 7 days. After the incubation period, the tubers were examined for soft rot and tissue maceration symptoms.

The calculation was made using the visual assessment and disease severity (DS%), which refers to the ranking of each treated tuber depending on the scale from 0 to 5 (0 = no soft rot, 1 = 1–10% soft rot, 2 = 11–30% soft rot, 3 = 31–50% soft rot, 4 = 51–70% soft rot, and 5 = > 70% soft rot), using the formula mentioned by Elhalag et al.^53^:

12 $$DS~\left( \% \right)=~\frac{{\sum \left( {Infection~grading~ \times ~~EquationNumber~of~tubers~representing~each~\inf ection~~grade} \right)}}{{\left( {total~~EquationNumber~of~tubers~ \times highest~\inf ection~grade~} \right)}} \times 100~~~$$

The treatment efficiency (TE%) was estimated as follows:

13 $$TE~\left( \% \right)~=~\frac{{\left( {DS~in~the~positive~control~group - DS~in~treatment~} \right)}}{{\left( {DS~in~the~positive~control~group} \right)~}}~ \times 100$$

### MP UV-C reactor power and specific energy consumption

The total electric power consumption (active power) of a UV-C reactor, including that of the lamps, ballasts, pump, and control and monitoring units, was calculated as recommended by NYSERDA UV System Cost Analysis Tool (UVCAT)^54^. It was also measured using the El Sewedy Electrometer during the biodosimetry tests conducted at flow rates of (25, 30, 35, and 40 m3/h) and lamp powers of (150, 210, 270, and 330 W) under a certain UVT of 85%. The specific energy consumption (SEC) is defined as a measure of power efficiency. It represents the electrical power consumption, P, necessary to disinfect one cubic meter of irrigation water at a certain UVT, corresponding to the minimum D_val_. It can be calculated as the ratio of the electrical power consumption P expressed in watts and the maximum flow rate (Q) in cubic meters per hour:

14 $$\:\mathrm{S}\mathrm{E}\mathrm{C}\:\left(\mathrm{U}\mathrm{V}\mathrm{T}\right)\:=\:\frac{\mathrm{P}}{\mathrm{Q}\:\left(\mathrm{U}\mathrm{V}\mathrm{T}\right)}$$

The SEC (UVT) is expressed in watt-hours per cubic meter (W.h/m^3^). The calculated SEC values were later used to evaluate the energy efficiency of different lamp configurations and to identify the most cost-effective operating condition for achieving at least a 4-log reduction in bacterial counts.

### Cost analysis and economic evaluation

The cost analysis was calculated according to Oida^55^ with modifications made using the UV System Cost Analysis Tool (UVCAT)^54^. To evaluate the financial viability of the proposed MP UV-C reactor, three parameters were computed and analyzed. These parameters are the MP UV-C reactor operating cost, the net present value (NPV), and the payback period (PBP). The operating costs are calculated using Eq. (15).

15 $$\:{\mathrm{T}}_{\mathrm{c}}=\frac{\mathrm{D}\:+\mathrm{I}+\mathrm{T}\mathrm{H}\mathrm{I}+\:\mathrm{R}+\mathrm{E}+\:{\mathrm{C}}_{\mathrm{c}\:}+{\mathrm{L}}_{\mathrm{c}}\:}{{\mathrm{n}}_{\mathrm{h}}}\:$$

Where: Tc = Total cost (L.E./h); nh = Annual working hours = 2400 (hr/year); D = depreciation; I = annual capital interest taxes; THI = housing and insurance cost; R = maintenance and repair cost (as recommended by UVCAT); E = electric power cost; Cc = chemicals for UV lamps cleaning cost; Lc = labor cost for operating the reactor.

Calculation NPV:

NPV was calculated using the general Eq. (16):

16 $$\:\mathrm{N}\mathrm{P}\mathrm{V}=\sum\:\left[\frac{\mathrm{C}\mathrm{a}\mathrm{s}\mathrm{h}\:\mathrm{f}\mathrm{l}\mathrm{o}\mathrm{w}\:\_\:\mathrm{t}}{{\left(1+r\right)}^{t}}\right]\:\:-\mathrm{I}\mathrm{n}\mathrm{i}\mathrm{t}\mathrm{i}\mathrm{a}\mathrm{l}\:\mathrm{I}\mathrm{n}\mathrm{v}\mathrm{e}\mathrm{s}\mathrm{t}\mathrm{m}\mathrm{e}\mathrm{n}\mathrm{t}\:$$

Where: Cash flow_t: The net cash flow for a specific period (t); r: The discount rate (representing the cost of capital or required rate of return) = 11%; t: The time period (e.g., year 1, year 2, etc.); Initial Investment = upfront cost of the project (typically a negative value as it’s an outflow) = 86,250 L.E.

NPV for UV-C Reactor was calculated assuming a Lifespan: 5 years.

Calculating the PBP for UV-C Reactor:

17 $$\:\mathrm{P}\mathrm{B}\mathrm{P}=\frac{\mathrm{I}\mathrm{n}\mathrm{i}\mathrm{t}\mathrm{i}\mathrm{a}\mathrm{l}\:\mathrm{i}\mathrm{n}\mathrm{v}\mathrm{e}\mathrm{s}\mathrm{t}\mathrm{m}\mathrm{e}\mathrm{n}\mathrm{t}\:}{\mathrm{A}\mathrm{n}\mathrm{n}\mathrm{u}\mathrm{a}\mathrm{l}\:\mathrm{n}\mathrm{e}\mathrm{t}\:\mathrm{c}\mathrm{a}\mathrm{s}\mathrm{h}\:\mathrm{f}\mathrm{l}\mathrm{o}\mathrm{w}}$$

### Statistical analysis

A completely randomized design was used in all experiments. Statistical analysis of the UV-C reactor validation experiments was performed using Microsoft Excel (Microsoft Corporation, USA). Bacterial count reduction (log₁₀-transformed) for each pathogen was treated as the dependent variable, and UV dose (continuous, J/m^2^) and UVT (categorical, 75%, 85%, 95%) were entered as independent variables. Polynomial regression was performed by generating higher-order terms for UV dose (UVdose^2^, UVdose^3^) using the Compute Variable function. To test whether the effect of UV dose varied across UVT, an interaction term (UVdose × UVT) was included in the regression model. Model fit was evaluated using the coefficient of determination (R^2^), adjusted R^2^, and ANOVA. Significance of regression coefficients was assessed using p-values, with α = 0.05 as the threshold for statistical significance. Statistical analyses, including ANOVA and Tukey’s HSD test, were performed using SPSS Statistics v23 (IBM Corp., USA) at a significance level of 0.05 to compare the severity of soft rot in potato tubers caused by *P*. *carotovorum* with that observed under untreated SIW conditions. A post hoc Dunnett’s test (two-sided) was used to compare culturable bacterial counts in untreated water with those obtained at different UV doses. A non-parametric analysis (NPar tests, Mann–Whitney test) was conducted in SPSS 23 to compare the AUDPC values of tomato plants irrigated with UV-treated versus untreated water (Asymp. Sig., two-tailed). To evaluate the effects of lamp power and reactor flow rate on energy consumption, a univariate analysis of variance (ANOVA) was conducted, with lamp power and flow rate entered as fixed factors. Interaction effects between lamp power and flow rate were also examined. In addition, multiple linear regression analysis was performed to quantify the predictive contribution of lamp power and flow rate to energy consumption, with regression coefficients (B), standard errors (SE), standardized coefficients (β), t-values, and significance levels reported. Post-hoc comparisons were carried out using Tukey’s HSD test to identify pairwise differences among lamp power and flow rate levels. Statistical significance was set at *p* < 0.05 for all analyses.

## Results

### Surface irrigation water analysis

Physico-chemical analyses were conducted to establish the baseline characteristics and determine the design requirements of the proposed UV-C disinfection system. The results revealed moderate levels of turbidity and TSS, while UVT was below the ideal range, indicating the necessity of pre-filtration to enhance UV penetration. EC and TDS were within acceptable irrigation limits but slightly above the optimum range for UV disinfection. Passing the river-sourced irrigation water through a 5-micron filter significantly improved water quality, reducing turbidity from 7.82 to 2.7 NTU, lowering TSS, and increasing UVT from 75% to 85%, placing these parameters within the recommended range for efficient UV-C performance. Decreases were also observed in COD and TDS, while pH, alkalinity, and heavy metals remained within recommended limits. Supp. Table 6 summarizes the results obtained for both unfiltered and filtered SIW samples, along with the recommended values for effective UV-C disinfection.

Both *R. solanacearum* and *P. carotovorum* were below the detection limit in the collected irrigation water.

### UV dose–response curves

**Fig. 2:**
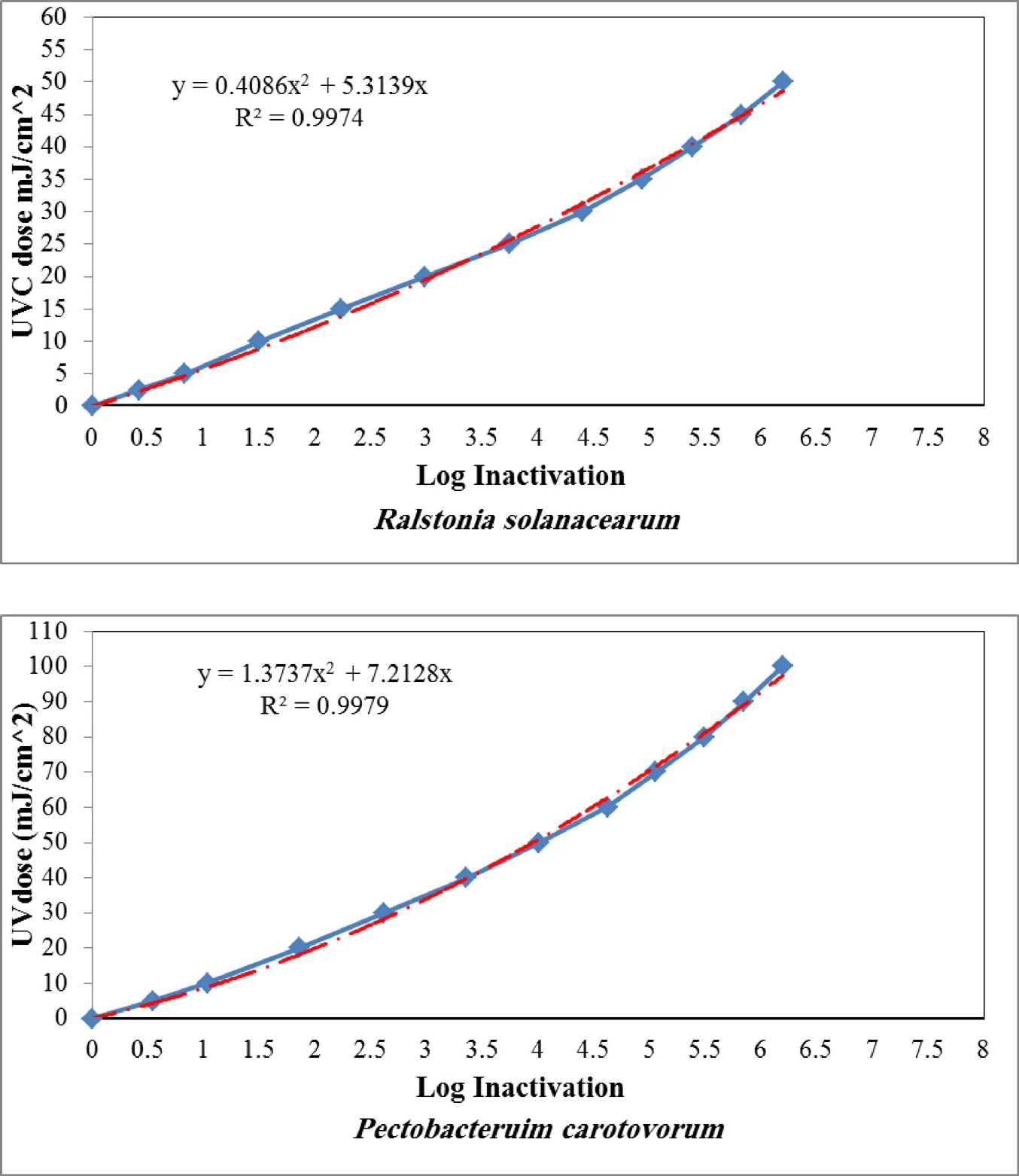
UV dose–response curves (DRCs) for *R*. *solanacearum* and *P*. *carotovorum* developed from collimated beam tests. Log inactivation: reduction in pathogen counts, expressed as log_10_ CFU/mL of water, in response to different UV doses.

To establish the UV sensitivity of the target pathogens, UV DRCs were first developed using CB tests. The UV DRCs developed from the CB tests (Fig. 2) showed a strong polynomial relationship between the applied dose and the log inactivation for both target pathogens. For *R*. *solanacearum*, the best-fit regression was.

Dose (mJ/cm^2^) = 0.4086 (log I)^2^ + 5.3139 (log I), with R^2^ = 0.9974.

and for *P*. *carotovorum*.

Dose (mJ/cm^2^) = 1.3737 (log I)^2^ + 7.2128 (log I), with R^2^ = 0.9979.

Both equations exhibited excellent goodness-of-fit (R^2^ > 0.99), confirming that the applied UV dose and microbial inactivation followed predictable, nearly quadratic kinetics typical for bacterial UV inactivation curves.

Regression analysis confirmed the significant effects of UV dose and transmittance (UVT) on bacterial reduction for both R. solanacearum and P. carotovorum (Supp.Table 7). Higher doses and higher UVT levels resulted in greater log reductions, consistent with the dose–response curves shown in Fig. 2. A significant interaction between dose and transmittance further strengthened the decrease in pathogen survival, validating the trends observed in the DRC-based evaluation of the UV-C reactor.

### Reduction equivalent dose (RED) and validated dose (D_Val_) values

The CB-derived UV dose–response relationships were subsequently used to interpolate full-scale reactor inactivation data and derive the RED and D_Val_ values. The derived RED and D_Val_ values, obtained by interpolating full-scale reactor inactivation data onto the DRC equations, are summarized in Table 1. For *R*. *solanacearum*, RED values ranged from 2.76 to 27.79 mJ/cm^2^, while for *P*. *carotovorum*, RED values ranged from 3.95 to 50.83 mJ/cm^2^, corresponding to 0.5–4 log reductions. The calculated UV sensitivities showed an average of 6.23 mJ/cm^2^/log for *R*. *solanacearum* and 10.30 mJ/cm^2^/log for *P*. *carotovorum*, indicating that *R*. *solanacearum* was more easily inactivated and required lower UV doses to achieve equivalent levels of inactivation. For instance, *R*. *solanacearum* needed about 27.79 mJ/cm^2^ to go down by 4 logs, while *P*. *carotovorum* needed about 50.83 mJ/cm^2^. Thus, *R*. *solanacearum* was less resistant to UV-C irradiation than *P*. *carotovorum*. The ratio between the RED and D_Val_ was consistently 0.88 for both pathogens, indicating that the dose measured in the CB setup was approximately 12% higher than the actual dose received in the reactor. This difference arises mainly from the applied correction factors (Petri, reflection, uncertainty of the UV dose-response fit, UV sensors calibration, and water factors) and the geometry of UV exposure. Such deviation (< ± 20%) complies with international validation limits, confirming the reactor’s reliable delivery of the intended UV dose. The average D_Val_ values demonstrated that *P*. *carotovorum* required nearly 1.64 times higher UV dose than *R*. *solanacearum* to achieve the same log reduction level, reflecting its greater resistance to UV-C radiation. These validation results confirm that the reactor delivered UV doses consistent with the requirements determined from DRC testing, thereby validating its performance for both pathogens.

### Optimization of UV-C reactor performance

**Fig. 3:**
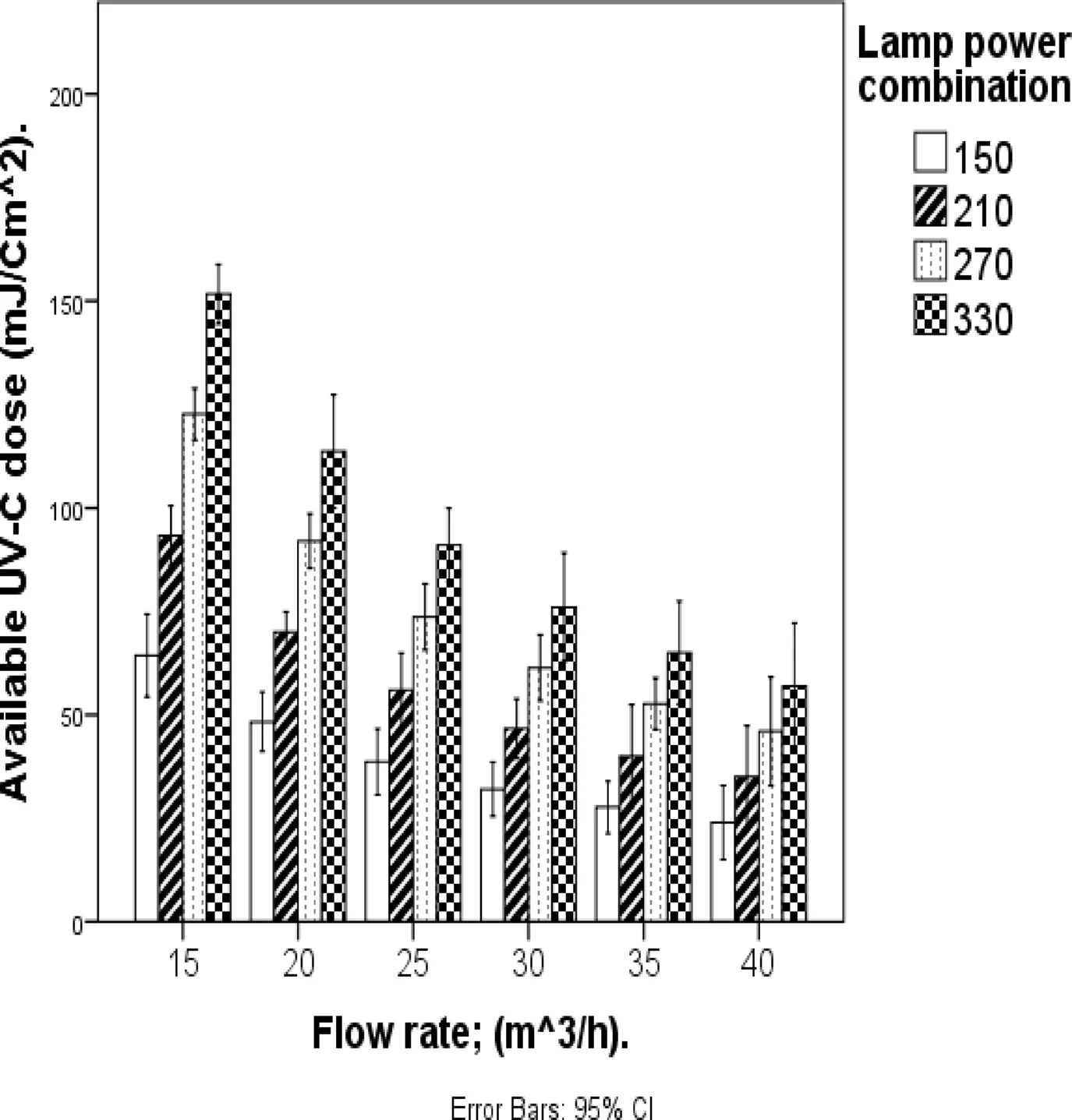
Effect of flow rate and total lamp power (W) on the available UV-C dose in the multi-processor UV-C reactor for *R*. *solanacearum* and *P*. *carotovorum* inactivation. The available dose decreased progressively with increasing flow rate (F = 515, *P* < 0.01), reflecting the reduced hydraulic residence time and UV exposure within the reactor. There was a significant interaction between flow rate and lamp power combination (F = 15, *P* < 0.01).

Following the validation of the RED for *R*. *solanacearum* and *P*. *carotovorum*, reactor optimization was conducted to identify the most energy-efficient configuration capable of delivering the required UV dose (D_val_ = 0.88 × RED) at the lowest power consumption. Table 2; Fig. 3 illustrate the relationship between the available UV-C dose and the flow rate under four total lamp power combinations (150, 210, 270, and 330 W). As expected, the available dose decreased progressively with increasing flow rate (F = 515, *P* < 0.01), reflecting the reduced hydraulic residence time and UV exposure within the reactor. At the lowest flow rate (15 m^3^/h), the available UV-C dose ranged between 64.27 and 151.76 mJ/cm^2^ (*P* < 0.01), whereas at 35 m^3^/h it declined to 27.54–65.04 mJ/cm^2^ (*P* < 0.01), depending on the lamp combination. There was a significant interaction between flow rate and lamp power combination (F = 15, *P* < 0.01). At a flow rate of 40 m³/h, no significant differences were observed in the effect of lamp power combinations between 150 W and 210 W, 210 W and 270 W, or 270 W and 330 W on the available UV-C dose. A UV dose of 52.54 mJ/cm^2^, achieved at a total lamp power of 270 W and flow rate of 35 m^3^/h, was sufficient to exceed the required inactivation thresholds for both *R*. *solanacearum* (24.38 mJ/cm^2^ after RED correction) and *P*. *carotovorum* (44.59 mJ/cm^2^). Therefore, this configuration (270 W at 35 m^3^/h) was identified as the optimal operating condition, providing effective 4-log microbial reduction while maintaining favorable energy efficiency for continuous field application.

### Effect of irrigation water treatment by using the MP UV-C-reactor on culturable microbial diversity

**Fig. 4:**
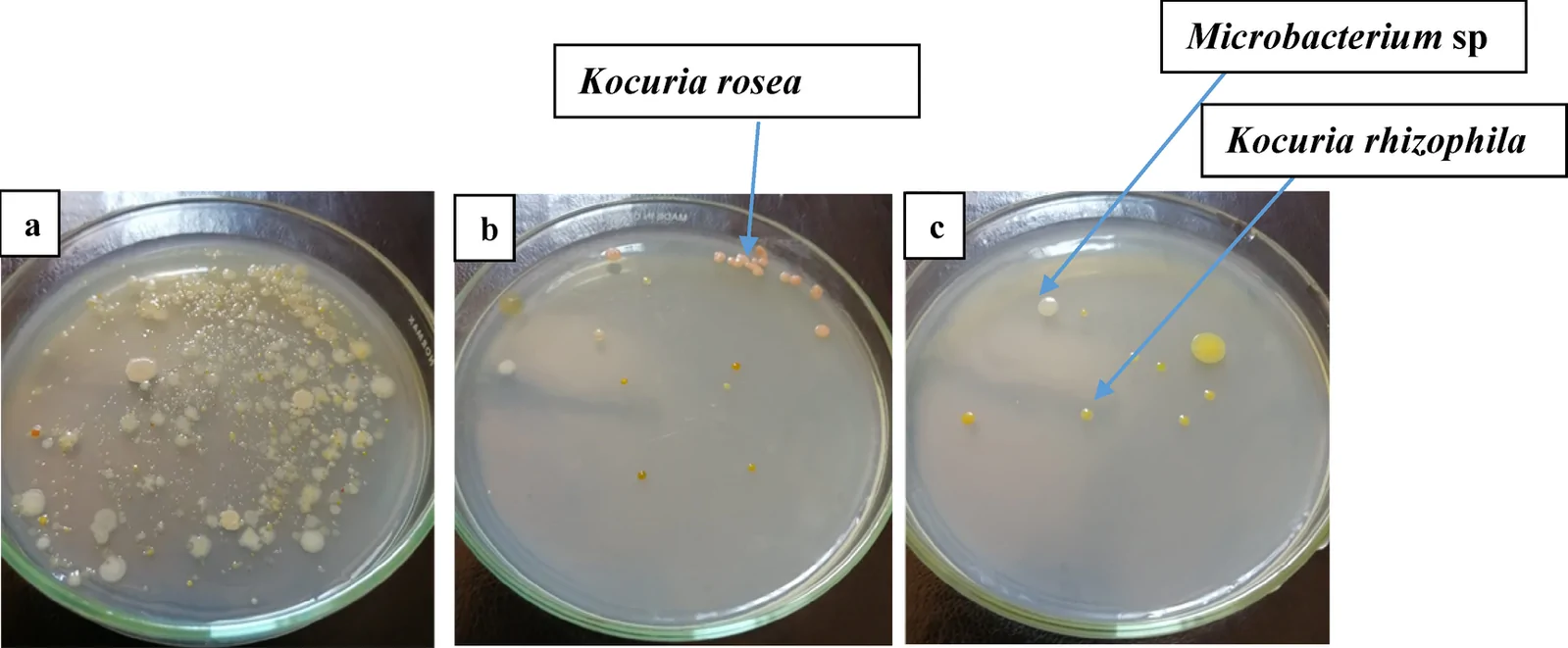
Count of bacterial colonies in surface irrigation water (SIW). (**a**): Control SIW was passed through the multi-processor UV-C reactor (MP UV-C reactor) without filtration and without UV-C irradiation, (**b**): SIW treated with the MP UV-C reactor at optimum operating conditions (flow rate 35 m^3^/h and lamp power 270 W) at UVT of 75%. (**c**): SIW treated with MP UV-C reactor at optimum operating conditions (flow rate 35 m^3^/h and lamp power 270 W) at UVT of 85%. The UV-resistant bacteria were identified as: *Microbacterium* sp. strain A-UV (PP886040), *Kocuria rosea* strain E-UV (PP886729), and *Kocuria rhizophila* strain D-UV (PP886128).

Culture-based results confirmed a marked decline in the total bacterial load of the SIW following UV-C treatment under optimal operating conditions (flow rate: 35 m^3^/h, lamp power: 270 W). As shown in Supp. Table 8, bacterial counts decreased from 4.47 ± 0.01 to 2.36 ± 0.01 log_10_(CFU + 1)/ml at UVT 75% (UV-C only), and from 4.47 ± 0.01 to 1.68 ± 0.19 log_10_(CFU + 1)/ml at UVT 85% (combined filtration + UV-C), corresponding to reductions of 2.10 ± 0.01 and 2.88 ± 0.18 log_10_(CFU + 1), respectively. In contrast, filtration alone (UVT 85%) caused only a minor reduction from 4.47 ± 0.01 to 4.37 ± 0.01 log_10_(CFU + 1)/ml (0.10 ± 0.00 log_10_(CFU + 1)), and the untreated control showed no significant change (4.47 ± 0.01 log_10_(CFU + 1)/ml). Significant differences were observed between the UV-C treatments and the control (*P* ≤ 0.002), whereas filtration alone did not differ significantly from the control. The visual count of colonies (Fig. 4) reflected a clear reduction in bacterial density after UV exposure at both UVT levels. A few colonies persisted after treatment and were identified as *Microbacterium* sp. strain A-UV (PP886040), *Kocuria rosea* strain E-UV (PP886729), and *Kocuria rhizophila* strain D-UV (PP886128). Most of the recovered bacterial species are characterized by clear colony pigmentation.

### Effect of UV dose on *R. solanacearum* cell wall

**Fig. 5:**
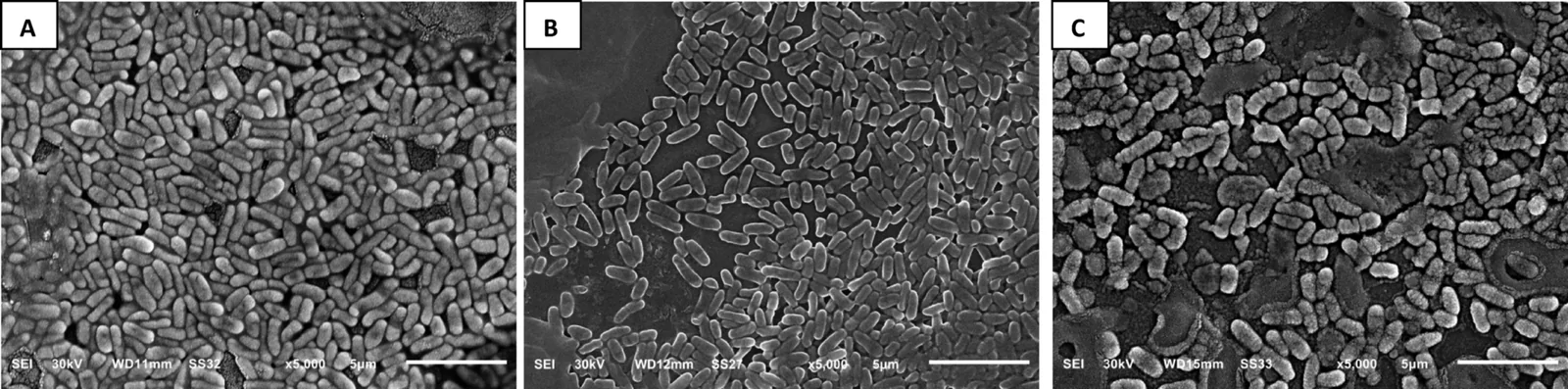
Scanning electron microscopy (SEM) images for ***R***. ***solanacearum*** bacterial cells in treated water by UV-C. (**A**): Control (untreated bacterial cells) (normal cell wall). (**B**): Bacterial cells treated with UV-C at 25 mJ/cm^2^ (shrunken cell). (**C**): Bacterial cells treated with UV-C at 50 mJ/cm^2^ (Cell wall appears abnormal and damaged). Samples were coated with gold-palladium membranes and examined using a Jeol JSM-6510 L.V SEM. The SEM operated at 30 kV with a magnification of 5000×. The stage was positioned at X = 12.860, Y = 3.839, Z = 14.000, T = 0.000, and *R* = 0.000, with a spot size of 33. S-BIAD3905 < Studies < BioStudies < EMBL-EBI.

*R*. *solanacearum* was chosen for this test because it showed greater sensitivity to UVC radiation than *P*. *carotovorum*. Figure 5 presents SEM images illustrating the morphological alterations in *R*. *solanacearum* cells exposed to UV-C irradiation. The untreated control (Fig. 5a) displayed typical rod-shaped bacterial cells with smooth, intact cell walls, indicating normal morphology and cellular integrity. Bacterial cells treated with UV-C at 25 mJ/cm2 (Fig. 5b) exhibited shrunken, suggesting sublethal damage caused by UV exposure. At a higher dose of UV-C (50 mJ/cm^2^), the morphological changes were more pronounced (Fig. 5c). The SEM images revealed extensive cell wall rupture and surface irregularities, confirming the severe damage induced by the higher UV dose levels. S-BIAD3905 < Studies < BioStudies < EMBL-EBI.

### Effect of irrigation water treatment by using the MP UV-C reactor on bacterial wilt

**Fig. 6:**
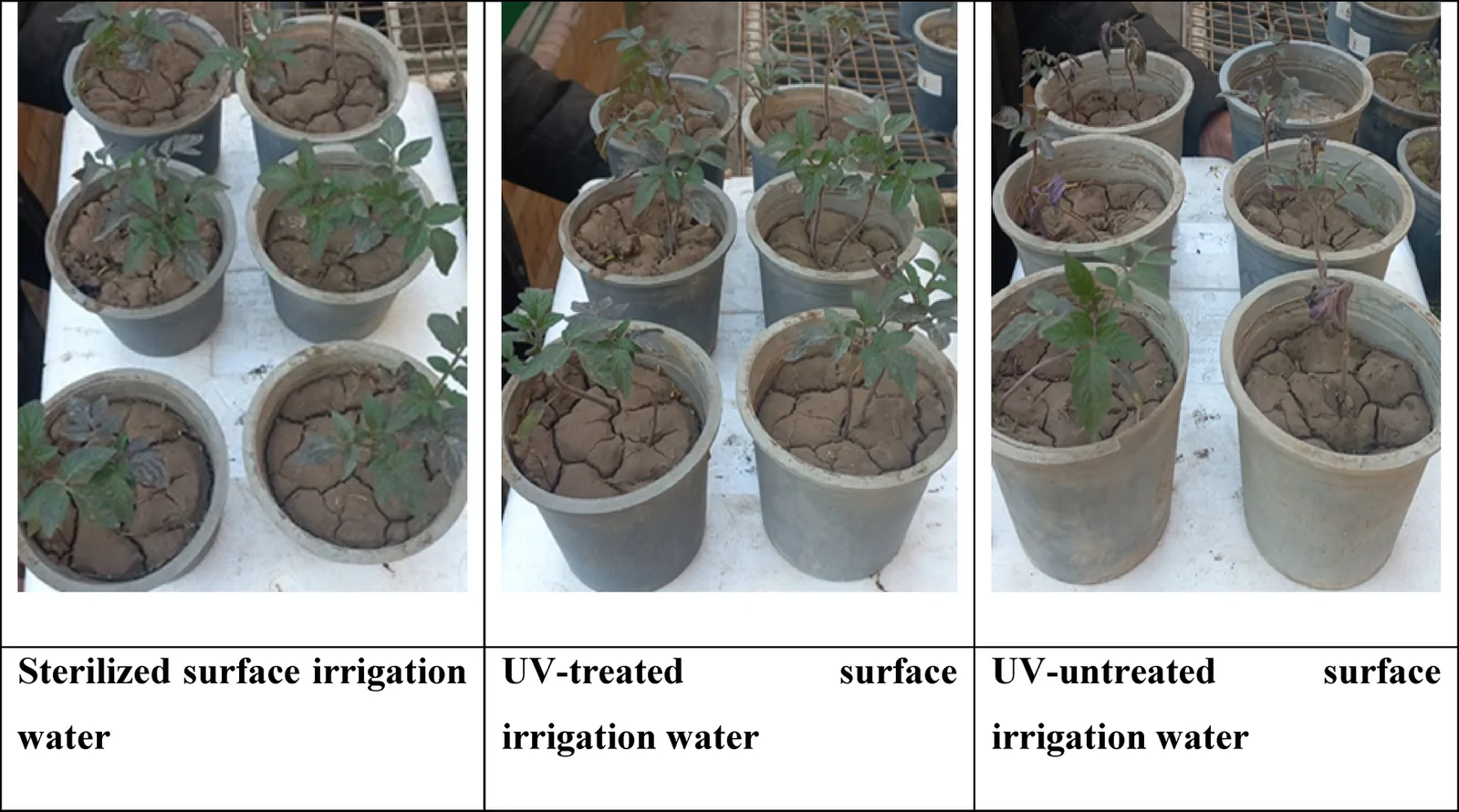
Effect of UV-C treatment of surface irrigation water (SIW) on bacterial wilt in tomato. Tomato plants (cv. Pinto, 2–3 weeks old) were inoculated with sterilized SIW (negative control), UV-C-treated SIW (52.54 mJ/cm^2^) using a multi-processor UV-C reactor, or untreated SIW contaminated with *Ralstonia solanacearum* at 10^8^ CFU/ml (positive control). UV-C treatment significantly reduced bacterial wilt compared to the positive control. The experiment duration was 32 days.

**Fig. 7:**
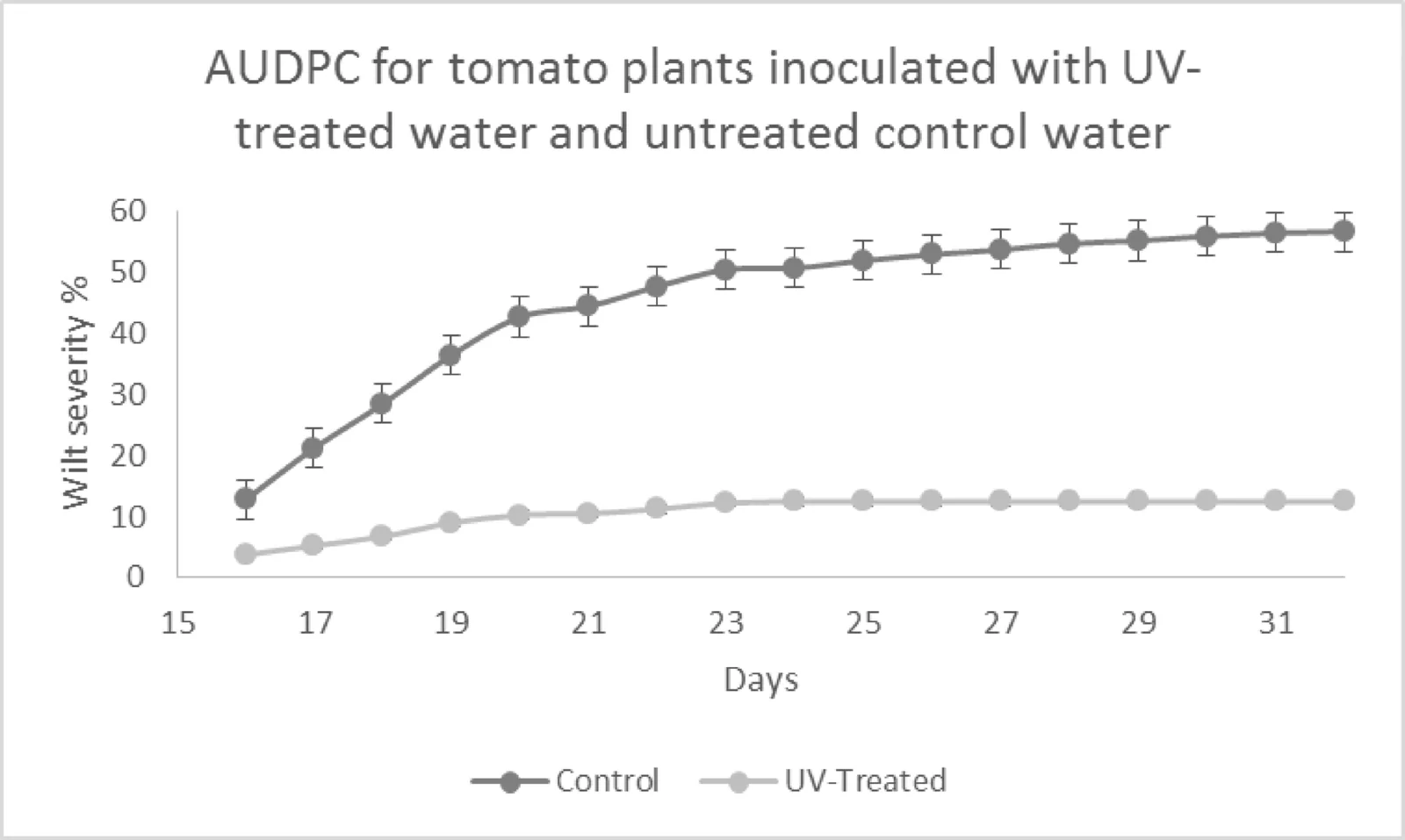
Area under the disease progress curve (AUDPC) was recorded daily based on the severity of wilt in tomato plants. Data represent the mean of 20 pots, each planted with two 3-week-old tomato seedlings (Experiment 1). A significant decrease in AUDPC was observed with UV-treated surface irrigation water compared to non-treated water (*P* < 0.001).

Bioassay results demonstrated a significant suppression of bacterial wilt in tomato seedlings irrigated with SIW contaminated with *R*. *solanacearum* at 10^8^ CFU/mL after UV-C treatment (Fig. 6). The SIW was treated using the MP UV-C reactor with a UV dose of 52.54 mJ/cm², achieved at a total lamp power of 270 W and a flow rate of 35 m³/h. This suppression was evident when compared to seedlings irrigated with untreated SIW used as the positive control. No wilting symptoms appeared on the tomato plants in the negative control (sterile SIW). As shown in Fig. 7, AUDPC values were markedly lower in plants receiving UV-treated SIW throughout the experimental period. In experiment 1, AUDPC values decreased from 773 ± 108 (mean ± SD) for non-treated SIW to 182 ± 72 for UV-treated SIW (*P* < 0.001). The control plants exhibited rapid symptom development, reaching over 57% ± 8% wilt severity by day 32, whereas those supplied with UV-treated SIW maintained minimal symptoms, 13% ± 5% (*P* < 0.001). IFAS tests confirmed the AUDPC results, with no latent infection recorded at the end of the experiment. Experiment 2 showed a similar trend (data not shown). These results clearly indicate that the optimized MP UV-C reactor configuration effectively disrupts the pathogen transmission cycle and maintains SIW free of viable *R. solanacearum* cells, providing strong evidence for its practical application in controlling bacterial wilt disease under irrigation conditions.

### Effect of irrigation water treatment by using the MP UV-C reactor on bacterial soft rot

**Fig. 8:**
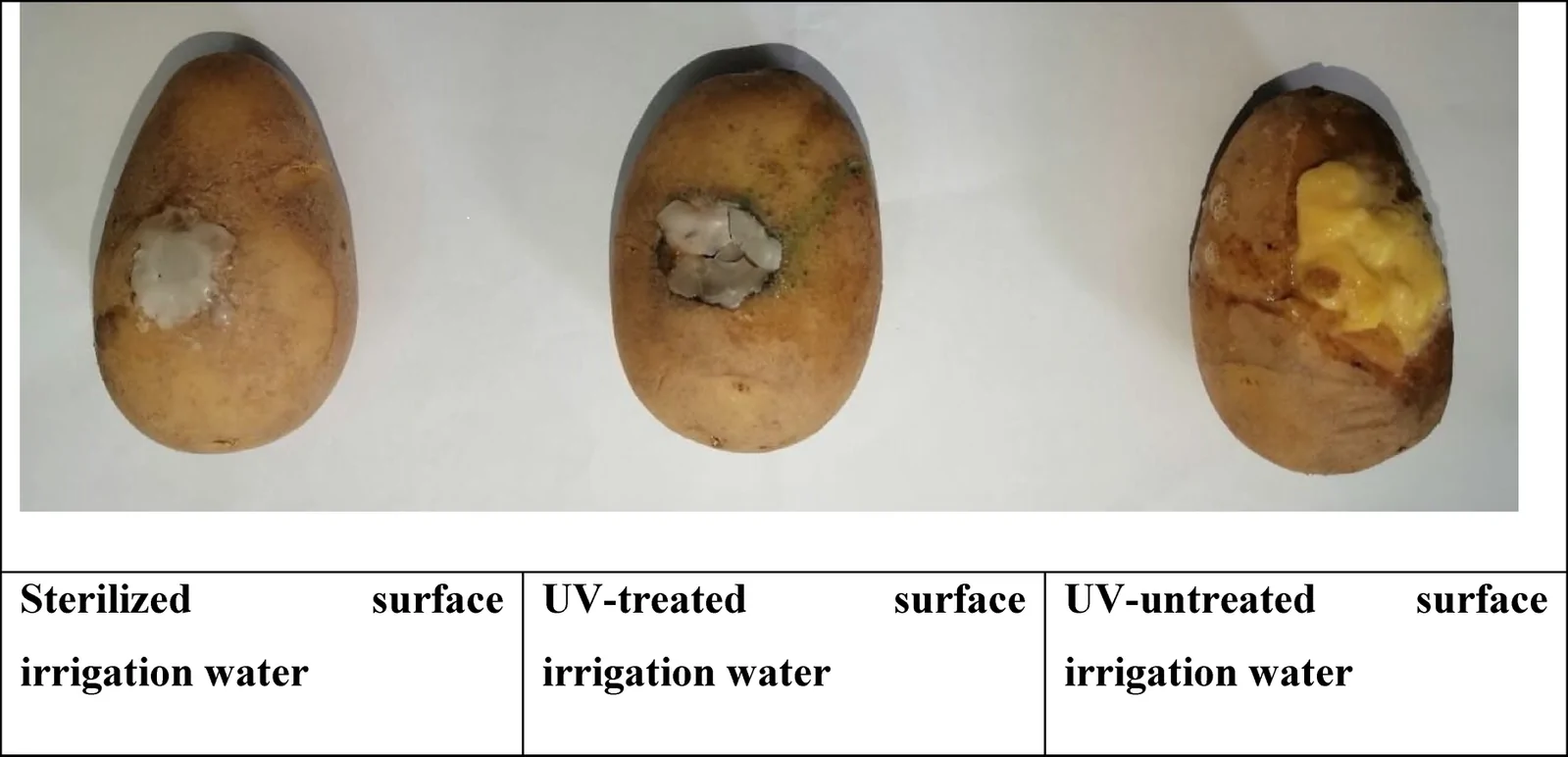
Effect of UV-C treatment of surface irrigation water (SIW) on bacterial soft rot in potato tubers. Tubers were inoculated with sterilized SIW (negative control), UV-C-treated SIW (52.54 mJ/cm^2^) using a multi-processor UV-C reactor, or untreated SIW contaminated with *Pectobacterium carotovorum* at 10^8^ CFU/ml (positive control). UV-C treatment significantly reduced soft rot compared to the positive control.

The obtained results showed that treating SIW (contaminated with *P*. *carotovorum* at 10^8^ CFU/mL) with the MP UV-C reactor (with a UV dose of 52.54 mJ/cm^2^, achieved at a total lamp power of 270 W and a flow rate of 35 m^3^/h) significantly reduced the severity of soft rot in potato tubers caused by *P*. *carotovorum* compared to the use of untreated SIW. As shown in Fig. 8, potato tubers inoculated with UV-treated SIW showed mild or no signs of rot, compared to the severe soft rot observed in those inoculated with untreated SIW. Furthermore, the negative control treatment (sterilized SIW) did not show any soft rot infection. Quantitative assessment revealed that using SIW disinfected with the MP UV-C reactor at the recommended dose led to a significant (*p* < 0.001) reduction in the severity of soft rot, achieving a DS value of 37.5 ± 1.4% and a TE of 62.5%. In contrast, the positive control group, which used untreated SIW, exhibited full disease development (DS = 100 ± 0.0%). Values expressed as means ± standard deviation (SD).

#### Specific energy consumption

Supp. Figure 5 illustrates the effect of flow rate and lamp power on the total electrical energy consumption of the proposed UV-C reactor when disinfecting irrigation water at 85% UVT. The highest power consumption (1.803 kWh) was recorded at a flow rate of 40 m³/h with a total lamp power of 330 W, whereas the lowest power consumption (1.0855 kWh) occurred at 25 m^3^/h and 150 W lamp power. Under the optimum operating conditions, with a flow rate of 35 m^3^/h and a total lamp power of 270 W (comprising 2 × 15 W + 2 × 30 W + 2 × 55 W + 1 × 70 W lamps), the reactor consumed 1.565 kWh. To evaluate energy performance, the specific energy requirement (SER) was calculated for each configuration (Supp. Figure 6). The SER values ranged between 48.82 Wh/m^3^ and 41.68 Wh/m^3^, depending on flow rate and total lamp power. The optimum SER value of 44.7 Wh/m³ was achieved under the optimum reactor configuration (270 W, 35 m³/h), which provided sufficient UV dose for complete disinfection of both pathogens while ensuring energy- and cost-effective operation. These results demonstrate that the MP UV-C reactor can deliver the required microbial safety standards for irrigation water with minimal power consumption.

Energy consumption (KWh) was significantly influenced by both lamp power and reactor flow rate. Descriptive analysis showed that energy consumption increased progressively with lamp power, ranging from the lowest values at 150 W to the highest at 330 W. Similarly, higher flow rates were consistently associated with higher energy consumption. Assumption checks confirmed that the data met the requirements for parametric testing. The Shapiro–Wilk test indicated normal distribution of residuals (*p* > 0.05). Univariate ANOVA revealed strong main effects of lamp power (F = 41.6, *p* < 0.001) and flow rate (F = 786.6, *p* < 0.001) on Energy consumption. Regression analysis further supported these findings. Lamp power was a significant predictor of specific energy consumption (B = 0.045, β = 0.222, t = 11.68, *p* < 0.001), as was flow rate (B = 0.194,, β = 0.976, t = 50.817 *p* < 0.001). Together, these predictors explained a substantial proportion of the variance in specific energy consumption. Post-hoc Tukey HSD comparisons clarified the differences among groups. Significant differences were observed in energy consumption across lamp power levels (*p* < 0.05) and across flow rates (*p* < 0.001).

Specific energy consumption (Wh/m^3^) was significantly influenced by both lamp power and reactor flow rate. Descriptive analysis showed that specific energy consumption increased progressively with lamp power, ranging from the lowest values at 150 W to the highest at 330 W. Conversely, higher flow rates were consistently associated with lower specific energy consumption. Assumption checks confirmed that the data met the requirements for parametric testing. The Shapiro–Wilk test indicated normal distribution of residuals (*p* > 0.05). Univariate ANOVA revealed strong main effects of lamp power (F = 318, *p* < 0.001) and flow rate (F = 129.9, *p* < 0.001) on specific energy consumption. A significant interaction effect was also observed (F = 310.6, *p* < 0.001), indicating that the influence of lamp power on specific energy consumption varied depending on the reactor flow rate. Regression analysis further supported these findings. Lamp power was a significant predictor of specific energy consumption (B = 0.024,, β = 0.830, t = 30.57, *p* < 0.001), as was flow rate (B = −0.182, β = −0.530, t = −19.4, *p* < 0.001). Together, these predictors explained a substantial proportion of the variance in specific energy consumption.

#### Operating cost of the MP UV-C reactor

Based on the operational cost model (Eq. 15), the estimated operating cost of the proposed MP UV-C reactor was approximately 60 L.E./h, equivalent to about 400 L.E./feddan. This demonstrates that the system can provide efficient and continuous disinfection of irrigation water at a relatively low and competitive cost while maintaining high energy performance and ease of operation.

#### Economic feasibility of the MP UV-C reactor

The total capital investment for constructing the reactor was 86,250 L.E. (2023 price level). With a rental value of 800 L.E./feddan, economic evaluation using cash-flow analysis yielded a Net Present Value (NPV) of 607,975 L.E. at an 11% discount rate, confirming strong profitability. Even when the discount rate was increased to 22%, the NPV remained positive, indicating financial robustness. The Payback Period (PBP) was only 0.46 years (≈ 6 months), signifying a rapid recovery of the initial investment.

Beyond direct financial returns, the reactor offers substantial agronomic and market benefits. It can disinfect irrigation water for approximately 366 feddans/year, each with an average potato yield of 15 tons/feddan, resulting in an annual production capacity of about 5,490 tons. Assuming a conservative 10% reduction in disease-related losses, the net yield gain would reach 549 tons/year, corresponding to an additional 43.92 million L.E./year in revenue. Moreover, improving tuber quality and phytosanitary status enhances export potential. If 25% of the treated yield is directed to export markets, additional income could reach 19.6 million L.E./year, highlighting the reactor’s significant contribution to national agricultural profitability and sustainability.

## Discussion

The most significant challenge in controlling potato brown rot in the traditional Delta and Nile Valley regions is the use of contaminated irrigation water in certain districts^16,18^. Moreover, P. carotovorum represents a widespread problem associated with soft rot^38^. Irrigation water can also facilitate the dispersal of P. carotovorum and its infection of potato and other crops^56^.

SIW from Kafr Shukr village was tested and found to have chemical properties mostly suitable for irrigation. *R*. *solanacearum* and *P*. *carotovorum*, which cause bacterial wilt and soft rot in potatoes, respectively, are commonly found in SIW^17^. This highlights the importance of managing microbial contamination in irrigation water. Therefore, appropriate treatment plans are essential to ensure that irrigation water is free of microbes. UV-C sterilization has become an effective method for inactivating many bacteria, including both Gram-positive and Gram-negative bacteria^57^. The results of this study indicate a significant reduction in pathogens, including *R*. *solanacearum* and *P*. *carotovorum*, achieving a 4-log reduction in both pathogens, which is consistent with previous data indicating a decrease in the microbial load in river water after UV-C treatment^23^.

However, suspended particles in irrigation water can affect the effectiveness of UV-C by protecting microbes from exposure. This data demonstrates that pre-filtration or clarification is essential to increase UVT and overall disinfection efficiency^58^. The effectiveness of UV-C treatment depends not only on water quality but also on the design and setup of the UV-C system, as well as the UV flux used^59^. These factors are essential for optimizing disinfection steps and ensuring safe irrigation. In the current study, the UV disinfection unit’s filtration system, equipped with a set of filters to reduce irrigation water turbidity to levels suitable for UV disinfection. The filter set consisted of a screening filter, a disk filter, a microfiber filter, and finally a 5‑micron filter, ensuring maximum efficiency of ultraviolet radiation and minimizing deposits. Additionally, the turbulent flow of irrigation water within the unit prevents particle sedimentation.

Quartz sleeve fouling due to mineral precipitation is a recognized operational challenge in UV-C systems, as it can reduce UV transmittance and consequently affect the delivered UV dose. In the present study, the impact of fouling was limited due to the controlled experimental conditions and relatively short operation periods. Nevertheless, fouling and lamp aging factors were considered in reactor performance evaluation and economic analysis to better reflect realistic operation. In practical applications, mitigation strategies such as periodic cleaning, pre-filtration, and monitoring of UV transmittance (UVT) are essential to maintain stable system performance. Future developments of the proposed MP UV-C reactor will focus on integrating self-cleaning mechanisms to further enhance long-term operational reliability.

UV-C disinfection is a well-known method for inactivating many microorganisms, such as bacteria, viruses, protozoa, and fungi, in water. This method works through UV-C light (200–280 nm), which has bactericidal potential. Bacterial DNA absorbs UV photons best at wavelengths near 260 nm, producing pyrimidine dimers. These dimers impede DNA replication and transcription, leading to cell death. UV-C at wavelengths near 280 nm can also damage amino acids (phenylalanine, tryptophan, and tyrosine) in cell membranes and wall proteins. Damage to these proteins affects protein production and impairs repair enzymes, making microbes more susceptible to UV damage^60^. Validation results in this study show a strong polynomial correlation (Fig. 2) between UV dose and log inactivation for both *R*. *solanacearum* and *P*. *carotovorum* (R² > 0.99). This supports the expected UV inactivation of these pathogens. Previous research has shown that UV-C is effective in inactivating various pathogens, such as oomycetes (*Phytophthora* and *Phytopythium*) and bacteria (*Pseudomonas syringae* and *Clavibacter michiganensis*)^61,62^.

In this study, the RED values for *R*. *solanacearum* ranged from 2.76 to 27.79 mJ/cm², while *P*. *carotovorum* required higher doses (3.95 to 50.83 mJ/cm²) for reductions of 0.5 to 4 log (Table 1). Data indicate that *P*. *carotovorum* is more resistant to UV-C inactivation than *R*. *solanacearum*, possibly due to its stronger cell walls or its ability to repair DNA^63^. *R. solanacearum* is a Gram-negative β-proteobacterium with a thin peptidoglycan layer and a lipid-rich outer membrane. UV may cause oxidative stress, which can easily degrade its envelope (Fig. 5). The brittle envelope of *R. solanacearum*, which is easily broken down by plant defenses and antimicrobial molecules, was documented by Zhang et al.^64^, and Xue et al.^65^. Meanwhile, *P. carotovorum* (a Gram-negative bacterium from the Enterobacteriaceae family) has a more robust envelope structure and may create extracellular polysaccharides that provide some UV protection. For Enterobacteriaceae, Rcs, Cpx, Bae, and Psp pathways control envelope integrity and pathogenicity^66^.

In this study, a reactor setting of 270 W at 35 m³/h, which equals 52.54 mJ/cm², led to more than a 4-log decrease of *R. solanacearum* and *P*. *carotovorum* (Table 2; Fig. 3). This suggests that microbes can be killed at moderate UV levels. This aligns with earlier studies. For example, one study observed up to a 5-log drop in *P*. *carotovorum* and *Dickeya chrysanthemi* on produce using a 222 nm KrCl excimer lamp and a 280 nm UVC-LED, but at a higher UV dose of 450 mJ/cm². Even though the doses used are different, our data suggest that favorable flow and reactor design can significantly reduce microbes even when UV intensity is not high^67^. Additionally, another study highlighted that UV light can be attenuated by particles in the water, reducing its disinfection efficacy in hydroponic water. Their rotating advanced oxidation contactor (RAOC) addressed this issue by maintaining steady UV exposure, resulting in a reduction of *R*. *solanacearum* by over 2 logs^68^. Our reactor design also mitigates the effects of shading and scattering, ensuring consistent UV dose distribution and effective microbial eradication. The use of a set of filters down to 5 microns produces clear water, free from turbidity that causes shadowing and reduce the efficiency of UV doses.

In this research, *Kocuria* and *Microbacterium* showed UV resistance (Fig. 4); this finding aligns with previous studies conducted by Molina-Menor et al.^69^, and Reis-Mansur et al.^70^,. Most of the bacterial colonies recovered after UV treatment exhibited distinct pigmentation. UV-resistant species, such as *Kocuria* spp., are distinguished by their ability to produce protective pigments (e.g., carotenoids), possess efficient DNA repair mechanisms, and maintain robust defenses against oxidative stress^71,72^. In this work, SEM micrographs revealed different morphological alterations in *R*. *solanacearum* cells after UV-C irradiation (Fig. 5). UV-C radiation has been shown to significantly increase the proportion of cyclic fatty acids (*P* ≤ 0.05) in *Salmonella* serovars (*S*. *typhimurium*, *S*. *hadar*, and *S*. *zanzibar*). Furthermore, atomic force microscopy revealed that UV-C exposure caused morphological modifications and abnormalities on the *Salmonella* serovars bacterial cell surface, such as the formation of grooves and irregularities^73^.

In the present study, the optimized MP UV-C reactor (52.54 mJ/cm²) effectively inactivated *P*. *carotovorum* in irrigation water, significantly reducing soft rot in potato tubers (Fig. 8) by lowering the pathogen load delivered to tuber wounds, consistent with a source-reduction approach in plant pathology. This conclusion is in agreement with a study reported by Rocha et al.^74^, where a UV-C radiation dose of 34.5 kJ/m^2^ completely prevented the growth of *P*. *carotovorum* colonies under in vitro conditions, while in an *in planta* assay, fluorescent light showed greater effectiveness than UV-C radiation in suppressing soft rot development in potato tubers. Moreover, potato tubers exposed to fluorescent light accumulated higher amounts of glycoalkaloids compared to those treated with UV-C radiation. In our study, the MP UV-C reactor was less efficient in reducing the survival of *P*. *carotovorum* than that of *R*. *solanacearum*. However, it led to a moderate rate of reduction in soft rot incidence in tubers inoculated with UV-treated irrigation water (approximately 62.5%) in *in planta* assay conditions. The experiment involved a high bacterial concentration of *P*. *carotovorum* (10^8^ CFU/mL), deliberately wounded tubers, sealed incubation, and high humidity, all of which create a highly favorable environment for disease development and represent a worst-case scenario. Under typical field conditions, where pathogen concentrations are generally lower and environmental factors are less conducive to infection, the efficacy of UV treatment could be expected to be higher.

While many previous studies have focused on human-pathogen reduction in irrigation or reused water, our data also show utility against a major plant pathogen. In this context, a study by Jones et al.^32^, found that UV irradiation of surface water achieved about 99.9% inactivation of various plant-pathogenic bacteria and oomycetes in a liquid-processing unit that uses UV light for the decontamination of surface waters contaminated with different pathogens, i.e., *Escherichia coli*,* Listeria monocytogenes*,* Salmonella enterica*,* Clavibacter michiganensis subsp. michiganensis*,* Phytophthora capsici*, and *Pseudomonas syringae pv. tomato.*In agricultural irrigation systems, a study by Younis et al.^33^, demonstrated the use of a novel UV disinfection system to inactivate *Phytophthora ramorum*, the causal agent of Sudden Oak Death in California and Oregon. They concluded that the system showed about a 91.7% reduction of fungal counts. Another study reported by Ghimire et al.^62^, focused on assessing the efficacy of UV-C light for the inactivation of *Phytopythium* and *Phytophthora* species in water and concluded that UV-C (240 to 290 nm) light-emitting diode (LED) inactivated the tested pathogens.

From an energy and economic perspective, the optimized MP UV-C reactor demonstrated high efficiency at the recommended operating conditions. The relatively low SER, combined with moderate operating costs, supports its suitability for continuous on-farm application. Beyond direct cost considerations, the use of UV-treated irrigation water reduces pathogen pressure, lowers disease incidence, and improves crop quality, thereby enhancing phytosanitary compliance and export potential. Collectively, these findings indicate that the proposed MP UV-C reactor represents an energy-efficient and economically feasible tool for managing irrigation water quality and controlling potato bacterial diseases under realistic operational conditions.

This method is recommended only as part of an integrated disease management program—including early detection, enhancing soil suppressiveness through crop rotation, organic management and amendments, and application of antioxidants—rather than as a standalone strategy^50, 75, 76^.

## Conclusion

This study indicates that the MP UV-C reactor is effective in reducing bacterial contamination in SIW, with *R*. *solanacearum* showing greater sensitivity than *P. carotovorum*. Reactor performance was influenced by parameters such as lamp power and flow rate, and pre-filtration improved UVT, thereby enhancing disinfection efficiency. Overall, UV-C treatment represents a promising chemical-free approach for managing plant pathogenic bacteria in SIW, with potential benefits for mitigating plant pathogenic bacteria in SIW, with potential benefits for disease management, crop health, and food safety. However, the method is limited by the possible survival of sub‑lethal pathogen populations and the risk of biofilm formation, which should be considered in future applications.

## Supplementary Information

Below is the link to the electronic supplementary material.

Supplementary Material 1

## Data Availability

The datasets generated and/or analysed during the current study are available in the following repositories: Bacterial isolates: National Center for Biotechnology Information (NCBI), accession numbers [OR430097, OP603322, PP886040, PP886729 and PP886128].Electron microscopy data: Scanning electron microscopy (SEM) datasets supporting Fig. 5 have been deposited in the BioImage Archive (BioStudies) under deposition Accession: S BIAD3905, DOI: 10.6019/S BIAD3905.
