# Supplementary material for: Validation and optimization of a multi-processor UV-C reactor for inactivation of potato brown rot and soft rot pathogens in surface irrigation water in the Nile Delta
Source: Sci Rep. 2026 Sep 9;16:28197. doi: 10.1038/s41598-026-67661-0 (PMC13554111; doi:10.1038/s41598-026-67661-0)
Supplement: Supplementary file 1 [file 41598_2026_67661_MOESM1_ESM.docx]

|  |  |  |
| --- | --- | --- |
| **(a) MP UV-C reactor in the laboratory.** | **(b) MP UV-C reactor in the laboratory (side view).** | **(c) MP UV-C reactor in the field.** |

Supp. Fig. 1 Multi-processor UV-C reactor and its operation in the laboratory and field.

|  | **No.** | **Description** |
| --- | --- | --- |
|  | **1** | **Electronic Ballast** |
|  | **2** | **Set Screw** |
|  | **3** | **Quartz Compression Nut** |
|  | **4** | **UV Lamp** |
|  | **5** | **Quartz Dome Seal 2-211** |
|  | **6** | **Lamp O-ring 2-113** |
|  | **7** | **Quartz Dome Sleeve** |
|  | **8** | **Reaction Chamber** |
|  | **9** | **Lamp Extension Spring** |
|  | **10** | **Plastic Cap** |
|  | **11** | **Lamp Connector** |
|  | **12** | **3/4” MNPT In/Out Ports** |

Supp. Fig. 2 Exploded diagram of the UV-C processor and corresponding parts list.

Supp. Table 1 Technical specifications of the multi-processor UV-C reactor.

| Item | Specifications |
| --- | --- |
| Housing material | 304/316 Stainless Steel |
| Total UV Lamp (T5) power in all processors | 330 W (2X15+ 4X30 + 2X55 +70W) |
| Control unit | 9 surge protectors |
| Input voltage | 110/240 V, 50/60 Hz |
| UV-C Lamp | 9 (TUV-T5) UVC low-pressure germicidal lamp 4 pins in single ends without ozone, detailed in Supp. Table 2 |
| Water treatment capability | 40 m^3^/h |
| Water capacity | 30720 cm^3^ |
| Max. Operating Pressure | 10 bar |
| Test Pressure | 15 bar |
| Rated UV Dose at Rated Flow | up to 150 mJ/cm² @ 75% UVT |
| Electrical | 220-240 Volt AC / 50-60 Hz |
| Max. Operating Temperature | 40 °C |
| Power Consumption Overall | 408 VA @ 120 V, 428 VA @ 240 V |
| Ballast | 9 Green light germicidal Electronic Ballast (Part 518101 RH51-425-40L), with lamp failure alarm |
| UV Quartz sleeve | Open at both ends. 19mm OD and its length suit the UV-C lamp with each processor |
| O ring | O-rings suit Quartz sleeve with dimensions 21.73mm ID x 28.45mm OD x 3.36mm cross section |
| UV Intensity Monitor | (4 x Part # 4-UV/MS50-1/2 V3) |
| Solenoid Valve | (Part # 4-SV-2000-1) |
| Hour Meter | (Part # 4-HM-R100) (one each per lamp) |
| Plumbing | 3” MNPT In/Out |
| Pretreatment Sediment filter | Filter Housing with 5 CARTRIDGE 5 MICRON 75 m3/h |
| Electricity generator | 6 KW for operation in areas without access to an electrical grid |
| Water pump | 40 m3/h, withdrawal depth 6 m |
| Shipping Size | 140x140x150 cm |
| Weight | 138 Kg |

Supp. Table 2: Geometric dimensions of the housing, quartz sleeve, and UV-C lamp data specifications for each processor.

| Processor No. | Housing | | Quartz sleeve | | | Philips UV-C “TUV-T5” Germicidal lamp Specification | | | | | |
| --- | --- | --- | --- | --- | --- | --- | --- | --- | --- | --- | --- |
|  | Length (mm) | Diameter (mm) | Length (mm) | Diameter (mm) | Wall thickness (mm) | Power (W) | Diameter (mm) | Base length  (mm) | Voltage (V) | Current (mA) | Life (hour) |
| 1-2 | 865 | 50.8 | 855 | 19 | 1.5 | 15 | 16 | 850 | 47 | 410 | 8000 |
| 3-6 | 900 | 76.2 | 890 | 19 | 1.5 | 30 | 16 | 885 | 82 | 425 | 8000 |
| 7-8 | 940 | 76.2 | 930 | 19 | 1.5 | 55 | 16 | 925 | 120 | 512 | 8000 |
| 9 | 1115 | 76.2 | 1105 | 19 | 1.5 | 70 | 16 | 1100 | 190 | 600 | 8000 |

|  | **No.** | **Part name** |
| --- | --- | --- |
|  | **1** | **UV lamp housing** |
|  | **2** | **Low-pressure UV lamp** |
|  | **3** | **Collimating tube** |
|  | **4** | **Collimated beam** |
|  | **5** | **Petri dish** |
|  | **6** | **Inoculated sample** |
|  | **7** | **Stirring bar** |
|  | **8** | **Magnetic stirrer** |
|  | **9** | **Height-adjustable stand** |
|  | **10** | **UV-C light meter** |
|  | **11** | **Fan** |
|  | **12** | **Window and shutter** |

Supp. Fig. 3 Schematic diagram for the collimated beam apparatus.

Supp. Table 3 UV-C dose and corresponding exposure time in collimated beam tests for each pathogen.

| Treatment | Pathogen | | | |
| --- | --- | --- | --- | --- |
|  | *Pectobacterium carotovorum* | | *Ralstonia solanacearum* | |
|  | Applied dose (mJ/cm^2)^ | Exposure time (sec.) | Applied dose (mJ/cm^2^) | Exposure time (sec.) |
| 1 | 5 | 51 | 2.5 | 25 |
| 2 | 10 | 101 | 5 | 51 |
| 3 | 20 | 202 | 10 | 101 |
| 4 | 30 | 303 | 15 | 152 |
| 5 | 40 | 405 | 20 | 202 |
| 6 | 50 | 506 | 25 | 253 |
| 7 | 60 | 607 | 30 | 303 |
| 8 | 70 | 708 | 35 | 354 |
| 9 | 80 | 809 | 40 | 405 |
| 10 | 90 | 910 | 45 | 455 |
| 11 | 100 | 1012 | 50 | 506 |

Supp. Fig. 4 Block diagram of a biodosimetry test facility for full-scale multi-processor UV-C reactor testing.

Supp. Table 4 Experimental variables coding for biodosimetry test to validate and optimize multi-processor UV-C reactor performance.

| Experimental variables | Symbol | Value |
| --- | --- | --- |
| Lamp power combination ( W) | P_1_ | 150 |
|  | P_2_ | 210 |
|  | P_3_ | 270 |
|  | P_4_ | 330 |
| Flow rate ( m^3^/h) | Q_1_ | 25 |
|  | Q_2_ | 30 |
|  | Q_3_ | 35 |
|  | Q4 | 40 |

Supp. Table 5 Wattage and number of lamps in each tested power combination of multi-processor UV-C reactor.

| Lamp type | 15W | 30W | 55W | 70W | Total power (w) |
| --- | --- | --- | --- | --- | --- |
| Treatments | No. of lamp | | | |  |
|  | 2 | 4 | 2 | 1 |  |
| P1 | 30 | 120 | -- | -- | 150 |
| P2 | 30 | -- | 110 | 70 | 210 |
| P3 | 30 | 60 | 110 | 70 | 270 |
| P4 | 30 | 120 | 110 | 70 | 330 |

Supp. Table	6 Physico-chemical characterization of surface irrigation water (unfiltered and filtered) and recommended values for UV-C disinfection.

| Characterization | | Surface irrigation water* | | | |
| --- | --- | --- | --- | --- | --- |
|  |  | Unit | Unfiltered | Filtered | **Recommended value |
|  |  |  | Average | Average |  |
| PH | | --- | 7.7 | 7.31 | 6.5 – 8.5 |
| Turbidity | | NTU | 7.82 | 2.7 | ≤ 5 for UV systems |
| UV Transmittance (UVT) | | % | 75 | 85 | ≥ 80 for UV systems |
| Total organic carbon (TOC) | | mg/L | 7.4 | 7.2 | < 10 |
| Chemical oxygen demand (COD) | | mg/L | 10 | 7 | < 30 |
| Electrical conductivity (EC) | | µS/cm | 412 | 404 | < 750 |
| Total dissolved solids (TDS) | | mg/L | 351 | 308 | < 450 |
| Total suspended solids (TSS) | | mg/L | 5.7 | 4.3 | < 10 |
| Total Alkalinity mg/L CaCO_3_ | | mg/L CaCO₃ | 43 | 41 | < 100 |
| Total Hardness | | mg/L CaCO_3_ | 70 | 54 | < 100 |
| Heavy metals conc. | Iron (Fe) | mg/L | 0.03 | 0.03 | < 0.3 |
|  | Manganese (Mn) | mg/L | 0.035 | 0.03 | < 0.1 |

* Surface irrigation water from the watercourse in Kafr Shukr, Qalyubia. ** Based on previous studies.

Supp. Table 7 Regression analysis of UV dose and UV transmittance on bacterial reduction.

| Pathogen | Predictor | B | T | p-value |
| --- | --- | --- | --- | --- |
| *R. solanacearum* | Intercept | -1.20 | -13.40 | <0.01 |
| *R. solanacearum* | UV dose | +0.14 | 25.43 | <0.001 |
| *R. solanacearum* | UVT | +1.49 | 15.11 | <0.001 |
| *R. solanacearum* | UV dose × UVT | (Interaction) | - | <0.001 |
| *P. carotovorum* | Intercept | -1.26 | -13.46 | <0.01 |
| *P. carotovorum* | UV dose | +9.65 | 33.00 | <0.001 |
| *P. carotovorum* | UVT | +1.58 | 15.36 | <0.001 |
| *P. carotovorum* | UV dose × UVT | (Interaction) | - | <0.001 |

Supp. Table 8 Total bacterial count of irrigation water treated with MP UV-C reactor at optimum operating conditions (flow rate 35 m^3^/h and lamp power 270 W).

| Treatment | UVT(%) | Total bacterial count (log_10_(CFU+1) ± SE)/ml | | Bacterial density reduction (log_10_) |
| --- | --- | --- | --- | --- |
|  |  | Before | After |  |
| Filtration only (5 µm) | 85 | 4.47±0.01 | 4.37±0.01 | 0.10±0.00a |
| UV-C only | 75 | 4.47±0.01 | 2.36±0.01 | 2.10±0.01b |
| Filtration + UV-C | 85 | 4.47±0.01 | 1.68±0.19 | 2.88±0.18c |
| Control (no filtration, no UV-C) | 75 | 4.47±0.01 | 4.47±0.01 | 0.00±0.00a |

Similar letters indicate no significant difference, whereas different letters denote a significant difference (*P* ≤ 0.002).

Supp. Fig. 5 Effect of UV reactor flow rate and lamp (150, 210, 270, and 330 W) power on the energy consumption.

Supp. Fig. 6 Effect of UV reactor flow rate and lamp power(150, 210, 270, and 330 W) on the specific energy consumption.
